# *Oxalis corniculata* L. As a Source of Natural Antioxidants: Phytochemistry, Bioactivities, and Application Potential

**DOI:** 10.3390/antiox14111352

**Published:** 2025-11-11

**Authors:** Tao Zhong, Junying He, Hao Zhao, Chang Tan, Wenjing Zhou, Congming Wu, Jijun Kang

**Affiliations:** 1 Technology Innovation Center for Food Safety Surveillance and Detection (Hainan), Sanya Institute of China Agricultural University, Sanya 572025, China; sy20243051241@cau.edu.cn (T.Z.); sy20233051122@cau.edu.cn (J.H.); b20243050624@cau.edu.cn (H.Z.); tanchang@cau.edu.cn (C.T.); s20233050999@cau.edu.cn (W.Z.); wucm@cau.edu.cn (C.W.); 2 State Key Laboratory of Veterinary Public Health and Safety, College of Veterinary Medicine, China Agricultural University, Beijing 100193, China

**Keywords:** *Oxalis corniculata* L., phytochemistry, antioxidant activity, biological functions

## Abstract

*Oxalis corniculata* L. (*O. corniculata*) has attracted increasing attention as a natural source of antioxidants with diverse pharmacological potential. Phytochemical studies have identified a diverse spectrum of metabolites, dominated by flavonoids, polysaccharides, and organic acids. These compounds exhibit antioxidant properties as well as related biological activities, including anti-inflammatory, antimicrobial, neuroprotective, hypoglycemic, and anticancer effects. Its long-standing use in traditional remedies, along with its incorporation into approved Chinese patent medicines, underscores its safety and translational value. This review synthesizes recent advances in the chemical composition, bioactivities, and molecular mechanisms of *O. corniculata*, emphasizing its antioxidant-driven pharmacological prospects. The review highlights *O. corniculata* as a sustainable and accessible botanical resource with significant potential for the development of pharmaceuticals, dietary supplements, and health-promoting applications.

## 1. Introduction

Plant-derived natural products have long served as an indispensable source of bioactive compounds, owing to their structural diversity and broad pharmacological potential [1]. In the context of increasing demand for sustainable resources in food and health industries, herbs with wide adaptability, ease of cultivation, and abundant bioactive constituents are attracting renewed scientific and industrial interest [2,3].

*O. corniculata*, commonly known as creeping woodsorrel, belongs to the family Oxalidaceae and is one of the most widely distributed vascular plants worldwide [4,5]. Traditionally, it has been used in various medical systems to treat digestive, dermatological, and urinary disorders, and in modern practice it is also included in several approved Chinese patent medicines for related indications [6]. Pharmacological studies have demonstrated that extracts of *O. corniculata* exhibit diverse biological activities, including antioxidant, anti-inflammatory, antimicrobial, hypoglycemic, and anticancer effects [7]. Flavonoids, polysaccharides, and organic acids are recognized as the major classes of bioactive compounds in *O. corniculata* [8,9]. Building on these findings, subsequent studies suggest that these compounds play key roles in mediating its pharmacological effects through antioxidant enhancement and regulation of inflammation, metabolism, and cellular protection [10]. In addition, emerging formulation and delivery strategies, such as nanomaterial-based systems and exosome-mediated approaches [11], have been explored to improve stability, bioavailability, and functional efficacy. Preliminary toxicological evaluations in animal models also indicate a favorable safety margin, supporting the translational potential of *O. corniculata* [12].

Although extensive research has been conducted, a comprehensive and up-to-date synthesis of the phytochemistry, antioxidant mechanisms, and health-related applications of *O. corniculata* remains lacking. By systematically summarizing recent advances, this review aims to clarify its antioxidant basis, link bioactive constituents to biological activities, and provide new perspectives on its potential roles in dietary supplements and therapeutic applications. *O. corniculata* also offers a sustainable source for the development of natural antioxidant-based products.

## 2. Botanical Features and Geographic Distribution

**Figure 1:**
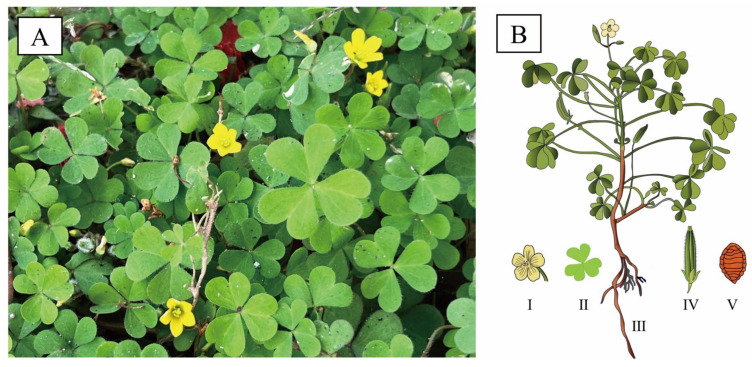
Morphology of *O. corniculata*. (**A**): Field photograph of *O. corniculata* displaying its characteristic trifoliate leaves and yellow flowers; (**B**): Diagrammatic illustration of *O. corniculata*, displaying the whole plant (III) with detailed depictions of the flower (I), leaf (II), fruit (IV), and seed (V).

*O. corniculata* is a small herbaceous plant, annual to perennial in habit. It is characterized by profusely branched [13], slender, and creeping stems with reddish nodes that readily produce adventitious roots, enabling rapid vegetative propagation in environments (Figure 1) [14]. Plants typically reach 10–40 cm in height and are sparsely covered with fine hairs. The leaves are trifoliolate, bearing obcordate leaflets (4–16 mm long, 4–22 mm wide) notched at the apex and ciliate along the margins. Flowers are axillary, solitary, or borne in small umbels, each comprising five yellow petals (6–8 mm) and five lanceolate sepals (3–5 mm). The floral structure includes ten stamens of unequal lengths and a five-loculed ovary topped with capitate stigmas. The fruit is a cylindrical capsule (1–2.5 cm) that dehisces explosively at maturity, releasing ovoid seeds (1–1.5 mm) with transverse ridged-reticulate ornamentation [15]. Flowering and fruiting occur from March to September. (https://www.iplant.cn/, accessed on 1 September 2025)

**Figure 2:**
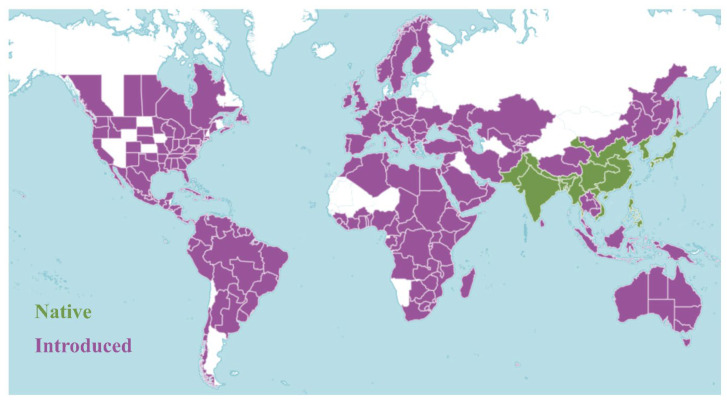
Global distribution of *O. corniculata*. Green areas indicate regions where the species is native, while purple areas represent regions where it has been introduced (https://powo.science.kew.org/taxon/urn:lsid:ipni.org:names:177893-2 (accessed on 1 July 2025)).

*O. corniculata* exhibits a nearly global distribution, thriving across tropical, subtropical, and temperate regions (Figure 2). Native to Asia, particularly East, South, and Southeast Asia, it has since naturalized across Europe, the Mediterranean, Oceania, and the Americas [16]. The species grows most vigorously at elevations of 500–1500 m, with higher density and frequency observed during the rainy season [17]. It commonly colonizes roadsides, grasslands, field margins, and forest edges, especially in moist or human-disturbed habitats. Its ecological success is largely attributed to adaptive growth habits and versatile reproductive strategies, which support survival and dispersal across diverse environments [18].

## 3. Phytochemistry

Phytochemical studies have identified 227 chemical constituents (Table S1) in *O. corniculata*, primarily isolated from its whole herb and leaves. Flavonoids are the predominant class of compounds, with approximately 84 identified in the plant [19], contributing to a total flavonoid content of up to 13.5 mg/g [10]. Organic acids, particularly phenolic acids, represent another major class of active constituents, with 51 organic acids identified, including 23 phenolic acids [20,21]. The total phenolic content in dry plant samples is quantified at 98.6 μg/g, with notable concentrations of hydroxybenzoic acid, p-coumaric acid, caffeic acid, and others [22]. The terpenoid fraction, primarily monoterpenes, consists of approximately 20 different compounds, with the highest concentration found in the leaves. Terpenoids such as geraniol and linalool have demonstrated antioxidant, antimicrobial, and anti-inflammatory activities [23].

**Figure 3:**
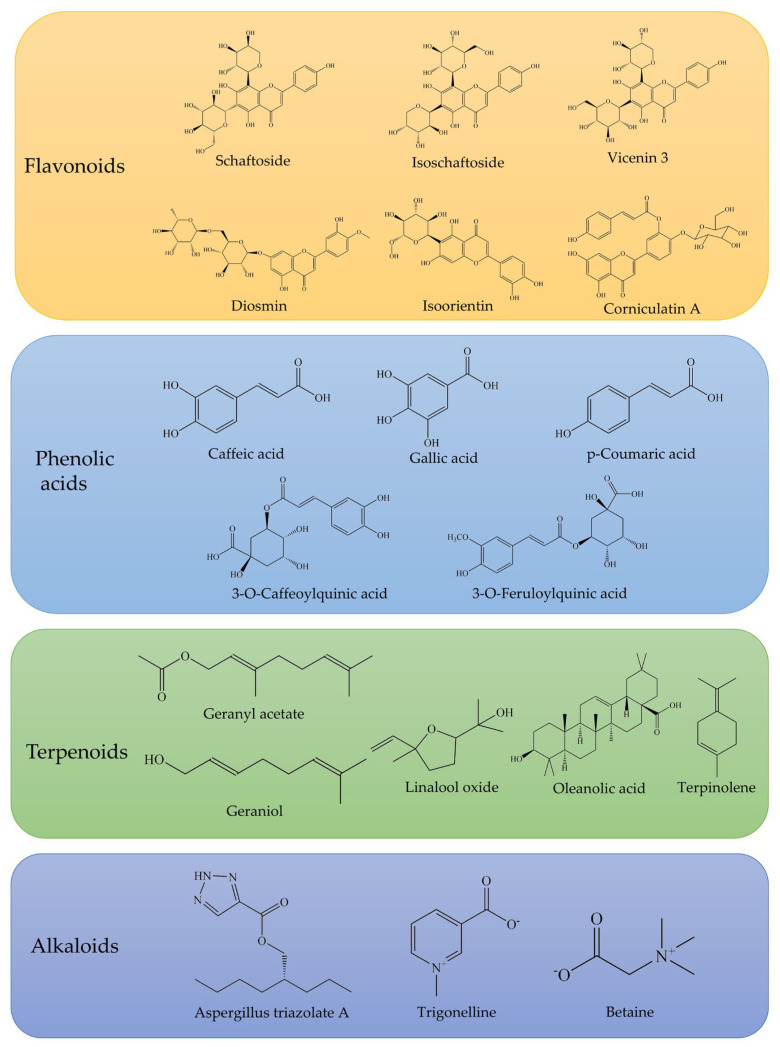
Representative flavonoids, phenolic acids, terpenoids, and alkaloids identified in *O. corniculata*.

Notably, compounds 47 and 48 (new flavones), compound 160 (a novel alkaloid, Aspergillus triazolate A), and compound 161 (a lignan named corniculin A) are among the novel constituents first isolated from *O. corniculata*. Additional constituents include coumarins, simple phenolics, fatty acid esters, anthraquinones, aldehydes, glycosides, sterols, carbohydrates, amino acids, carotenoids, pigments, and other specialized metabolites. Typical compounds are shown in Figure 3.

### 3.1. Flavonoids

Flavonoids are representative compounds in *O. corniculata*, featuring a basic 2-phenylchromen-4-one skeleton. They are categorized into several subclasses, including flavones, flavonols, dihydroflavones, isoflavones, chalcone, dihydrochalcones, and flavanols (Figures S1 and S2). The antioxidant activity of these compounds depends on both the number and position of hydroxyl groups in their structure. Hydroxylation at the 3, 5, and 7 positions and the presence of a C2=C3 double bond in the C-ring enhance radical-scavenging activity and anti-inflammatory effects [24]. For instance, flavonols possess a hydroxyl or other oxygen-containing substituent at the 3-position of the flavonoid backbone, a structural feature that has been associated with enhanced antioxidant activity [25]. In addition, glycosylation generally improves water solubility and bioavailability, which may enhance physiological efficacy [26]. These insights underscore the importance of structure-activity relationships in determining the functional properties of flavonoids.

In *O. corniculata*, flavonoid content may reach optimal levels when harvested in June or October, periods likely corresponding to peaks in secondary metabolite accumulation [27]. Flavonoids occur mainly as C-glycosyl and O-glycosyl flavones. C-glycosyl flavones (schaftoside, isoschaftoside, and isovitexin) demonstrate strong radical-scavenging activity and superior structural stability [28]. In contrast, O-glycosyl flavones (orientin, isoorientin and diosmin) exhibit enhanced solubility and absorption [29]. These flavonoids collectively represent the major contributors to the antioxidant and anti-inflammatory activities of *O. corniculata*.

### 3.2. Organic Acids

Organic acids constitute an important class of phytochemicals in *O. corniculata*, contributing both to its characteristic sour taste and to its diverse biological activities [20]. Among them, phenolic acids (85–107) are dominated by caffeic acid and its derivatives (Figure S3A). Caffeic acid content peaks in dry-area samples, reaching up to 1.28 μg/g [22], with lower concentrations observed in samples from marshy and moist areas. Caffeic acid derivatives, characterized by a catechol moiety, exhibit strong free radical scavenging capacity and protect cells against oxidative stress. Their antioxidant potential is largely determined by structural factors such as the number and position of hydroxyl groups on the aromatic ring, while modifications like methoxylation can further modulate activity [30].

In addition to phenolic acids, *O. corniculata* also contains low-molecular-weight organic acids such as malic and citric acids [20]. Fatty acid-type organic acids have been identified as well, with gas chromatography-mass spectrometry (GC/MS) analyses revealing oleic acid and 6-octadecenoic acid as predominant components (31.08%), followed by palmitic acid (2.55%) (Figure S3B) [31]. These fatty acids are associated with metabolic regulation and potential cardiovascular protective effects [32].

### 3.3. Terpenoids

Terpenoids, biosynthesized from isoprene units, constitute a structurally diverse class of natural products that contribute to plant defense, volatile signaling, and physiological regulation [33]. To date, 20 terpenoids (136–155) have been identified in *O. corniculata*, comprising monoterpenes (136–150), a cycloether monoterpene (151), and triterpenes (152–155).

Supercritical CO_2_ extraction followed by GC/MS analysis of leaf samples from Guangdong Province, China, revealed that monoterpenes dominate the essential oil fraction [23]. The most abundant constituents were geranyl acetate (13.3%), terpinolene (9.2%), linalool oxide (7.4%), and geraniol (6.4%). These compounds impart characteristic aromatic properties and exhibit antioxidant, antimicrobial, and anti-inflammatory activities [34]. In addition to volatile monoterpenes, several triterpenoids have been detected in the non-volatile fractions, including oleanolic acid, eburicoic acid, squalene, and phytol [31,35]. The chemical structures of the identified terpenoids are shown in Figure S4.

### 3.4. Alkaloids

Alkaloids are nitrogen-containing natural compounds with diverse chemical structures [36]. Five alkaloids (156–160) have been reported from *O. corniculata*. Among them, trigonelline (156), a classical pyridine alkaloid with an N^+^-methylated pyridine ring, displays high water solubility due to its quaternary ammonium structure [37]. Betaine (158), a naturally occurring zwitterion with a trimethylated amino group, serves as an important osmolyte and methyl donor, and has been associated with antioxidant, hepatoprotective, and metabolic regulatory effects [38].

Particularly noteworthy is compound 160, Aspergillus triazolate A (ATA), a novel triazole alkaloid first isolated from the dried whole plant of *O. corniculata*. Structurally, ATA consists of a hydrophilic triazole ring and a hydrophobic C_8_ alkyl chain, forming an amphiphilic scaffold. This structural balance is proposed to facilitate stable interactions with target enzymes, thereby enhancing inhibitory activity. Bioassays have demonstrated that ATA lowers blood glucose levels by inhibiting α-glucosidase (α-Glu), highlighting its potential as a natural lead compound for α-Glu inhibitor development [39]. The chemical structures of these alkaloids are shown in Figure S5.

### 3.5. Polysaccharides

Polysaccharides are natural macromolecules consisting of more than ten monosaccharide units linked through glycosidic bonds, often comprising hundreds to thousands of residues [40]. In *O. corniculata*, crude polysaccharides (OCP) are typically extracted using hot water and ethanol precipitation, followed by purification steps such as deproteinization and ethanol fractionation, with a total yield of 9.45% [7]. The major fraction, OCP-3, has a molecular weight of 31.5 kDa and is mainly composed of arabinose (47.83%) and galacturonic acid (17.81%), indicating its acidic nature. OCP-3 demonstrates strong free radical scavenging capacity, suppresses lipid peroxidation, and confers protection against oxidative damage in both cell-based and in vivo models [7].

### 3.6. Nutrients

On a dry-weight basis, *O. corniculata* contains considerable amounts of essential minerals, including calcium (5.63%), potassium (3.15%), and magnesium (2.63%) [41]. Trace elements are also present [41], with iron (89.16 ppm), manganese (4.21 ppm), zinc (1.59 ppm), and copper (0.12 ppm) detected. The vitamin C content reaches 0.139 mg/g. Fresh methanolic extracts provide approximately 710 μg vitamin C equivalent antioxidant capacity per gram of fresh leaves [42], reflecting strong antioxidant capacity attributable to vitamin C. This effect is likely enhanced by synergistic interactions with flavonoids, organic acids, and other bioactive constituents [43]. In addition, dried *O. corniculata* powder contains appreciable levels of crude protein (12.25%), crude fiber (10.64%), and ash (9.98%). The total antioxidant capacity (TAC) has been measured at 31.60 mmol ascorbic acid equivalent/kg dry weight and 31.22 mmol vitamin E equivalent/kg dry weight, indicating strong overall antioxidant potential [44].

## 4. Traditional Uses

*O. corniculata* has a long-standing history in traditional medicine, first documented in the Xin Xiu Ben Cao in 659 AD [45]. Over centuries, it has been incorporated into diverse ethnomedical systems, including Traditional Chinese Medicine (TCM), Ayurveda, and Unani. In oral applications, decoctions, teas, or fresh juices prepared from the whole plant or leaves are commonly used to relieve gastrointestinal discomfort, fever, urinary disorders, and liver ailments [6,46]. Topical preparations, such as poultices or expressed juices, are applied to treat skin conditions including warts, eczema, wounds, and abscesses, reflecting recognition of its antimicrobial and wound-healing effects [47,48].

In TCM, *O. corniculata* is traditionally categorized as a heat-clearing and detoxifying herb, used to eliminate damp-heat, relieve strangury, and reduce swelling and pain. It has been incorporated into several approved Chinese patent medicines, such as Mi Lin Qing Capsules (for damp-heat urinary tract infections/strangury), Fu Yan Xiao Capsules (for gynecological inflammation), and Gu Kang Capsules (for traumatic injury and swelling). These commercial formulations illustrate its compatibility within compound prescriptions and its translational potential from traditional use to standardized products. These applications reflect its traditional recognition for antimicrobial, antioxidant, and anti-inflammatory properties (Table 1).

## 5. Biological Activities of *O. corniculata*

**Figure 4:**
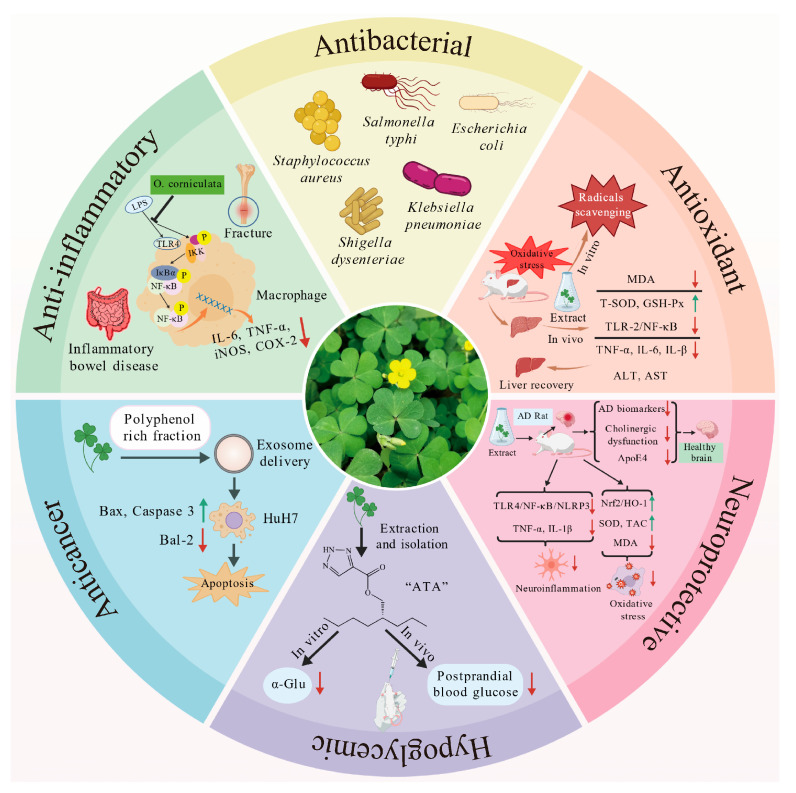
Biological Activities of *O. corniculata*. Upward-pointing arrows denote upregulation of gene/protein expression or protective markers, while downward-pointing arrows indicate downregulation of expression levels or damage markers. Several elements in the image were obtained from https://biogdp.com (accessed on 1 September 2025) [55].

*O. corniculata* exhibits a wide range of biological activities, increasingly supported by experimental evidence. These effects are largely attributed to the synergistic actions among its diverse phytochemicals, particularly flavonoids, polysaccharides, and phenolic acids. The plant demonstrates notable potential in antioxidant, anti-inflammatory, antimicrobial, hepatoprotective, neuroprotective, hypoglycemic, and anticancer applications. An overview of these biological activities is presented in Figure 4, providing a foundation for the detailed discussions that follow.

### 5.1. Antioxidant Activity

Oxidative stress caused by excessive reactive oxygen species (ROS) is a major contributor to lipid peroxidation, protein oxidation, and DNA damage, processes implicated in chronic diseases such as hepatic dysfunction, neurodegeneration, and cancer [56]. It suppresses enzymatic antioxidants, including superoxide dismutase (SOD), catalase (CAT), and glutathione peroxidase (GSH-Px), as well as non-enzymatic antioxidants such as glutathione (GSH). This imbalance leads to the accumulation of oxidative products such as malondialdehyde (MDA) and protein carbonyls (PC), which further aggravate cellular injury and inflammation [7].

*O. corniculata* contains abundant bioactive constituents, notably flavonoids, phenolic acids, and polysaccharides, that contribute to strong free radical scavenging and anti-oxidative stress effects (Table 2) [4]. The polysaccharide fraction OCP-3 inhibits lipid peroxidation and protects DNA from oxidative damage. In both HEK-293 cells and *C. elegans*, OCP-3 reduces MDA and PC levels, while enhancing antioxidant enzymes including SOD, CAT, and GSH-Px [7]. Methanol extracts of *O. corniculata* activate the Nrf2/HO-1 signaling pathway, enhancing antioxidant enzyme activities [57]. In carbon tetrachloride-induced hepatic and renal injury models, the extracts restore the activities of multiple antioxidant enzymes, including SOD, CAT, GSH-Px, glutathione-S-transferase (GST), glutathione reductase (GSR), peroxidase (POD), and quinone reductase (QR). The extracts also lower MDA levels, suppress lipid peroxidation, improve serum biochemical indices such as alanine aminotransferase (ALT) and aspartate aminotransferase (AST), and mitigate histopathological damage [58,59].

Dietary supplementation with *O. corniculata* powder has been shown to enhance antioxidant capacity under heat stress in broilers by increasing SOD, CAT, and GSH-Px activities while reducing MDA levels [60]. It also improved oxidative stability and nutritional quality of broiler meat by lowering lipid peroxidation and increasing polyunsaturated fatty acid (PUFA) content [4,5]. These findings indicate that *O. corniculata* supplementation effectively mitigates oxidative stress and maintains redox balance in animal systems, supporting its use as a natural antioxidant source in animal nutrition [44].

**Table 2:** Summary of antioxidant activity studies of *O. corniculata*.

No.	Testing Subjects	Application Part or Compounds	Doses/Duration	Effects	Ref.
1	Free radicals	Polyphenol-rich methanol extract	0.5–50 μg/mL	DPPH·scavenging	[61]
2	pUC18 plasmid DNA	Polyphenol-rich methanol extract	100–500 ng/mL	Protected DNA from·OH-induced strand breaks	[61]
3	BSA	Polyphenol-rich methanol extract	100–500 μg/mL	Inhibited PC formation	[61]
4	Liver cells	Polyphenol-rich methanol extract	10–50 µg/mL, 45 min incubation	Protected liver cells from oxidative damage by OH	[61]
5	Free radicals; Fe^3+^	Hot water extract	50–400 μg/mL	Free radicals scavenging and iron-reducing capacity	[62]
6	HEK-293 cells; *C. elegans*	Acidic polysaccharide OCP-3	50–800 μg/mL; 4–8 mg/mL	SOD ↑, CAT ↑, GSH-Px ↑, MDA ↓, PC ↓, radicals scavenging, DNA protection	[7]
7	Free radicals	Biogenic silver nanoparticles synthesized using aqueous extract of *O. corniculata*	50–400 μg/mL	DPPH·/ABTS·scavenging	[31]
8	Free radicals, Fe^2+^, Fe^3+^, lipid	Ethanol extract	DPPH: 26.2 ± 2 µg/mL; Ferrous Ion: 74.3 ± 0.4 µg/mL; NO: 75.4 ± 7.3 µg/mL; ABTS: 59.9 ± 5.2 µg/mL; NBT: 118.2 ± 2.3 µg/mL; FRAP: 152.1 ± 9.5 µg/mL; LPO: 1.8 ± 0.4 µg/mL	The antioxidant effect was as demonstrated by IC_50_ values in seven assays.	[63]
9	Free radicals	TFO	100–500 μg/mL	DPPH/OH/O_2_-scavenging	[10]
10	Pork lard	*O. corniculata* polyphenols	0.02–0.04%, 50 °C, 21 days	Significantly inhibited lipid peroxidation	[64]
11	Fractured rats	*O. corniculata* (whole plant, 47.5% of mixture) aqueous extract	150–600 mg/kg, percutaneous and p.o., 2 weeks	SOD ↑, CAT ↑, GSH ↑, enhances antioxidant enzyme activity and alleviates oxidative stress in bone tissue	[65]
12	AlCl_3_-induced AD rats	Methanol extract	150 mg/kg, p.o., 5 weeks	TAC ↑, SOD ↑, MDA ↓; upregulates Nrf2/HO-1 pathway, enhances antioxidant defense, reduces oxidative damage in brain	[57]
13	SD rats (CCl_4_-induced acute liver injury)	Aqueous extract	4–16 g/kg, p.o., 10 days	ALT ↓, AST ↓, MDA ↓, T-SOD ↑, GSH-Px ↑; downregulates TLR2/NF-κB pathway, inhibits oxidative stress	[66]
14	CCl_4_-induced nephrotoxic rats	Methanol extract	100, 200 mg/kg, p.o., 7 days	CAT ↑, POD ↑, SOD ↑, GSH-Px ↑, GST ↑, GSR ↑, QR ↑, GSH ↑, MDA ↓, protein oxidation ↓, renal injury markers ↓, improved renal histology	[58]
15	CCl_4_-induced hepatotoxic rats	Methanol extract	100, 200 mg/kg, p.o., 7 days	CAT ↑, POD ↑, SOD ↑, GSH-Px ↑, GST ↑, GSR ↑, QR ↑, GSH ↑, TBARS ↓, AST ↓, ALT ↓, ALP ↓, improved liver histology	[59]
16	Paracetamol-induced hepatotoxic rats	Ethanol extract	100–500 mg/kg, p.o., 4 days	AST ↓, ALT ↓, ALP ↓, MDA ↓, improved liver histology; enhanced antioxidant defense and reduced oxidative stress	[67]
17	Streptozotocin (STZ)-induced diabetic rats	Ethanol extract	100 and 300 mg/kg, p.o., 28 days	Reduced fasting glucose and MDA levels, enhanced SOD and GSH-Px activities, and improved pancreatic β-cell morphology.	[8]
18	Heat-stressed broilers	*O*. *corniculata* powder	10 g/kg diet, 28–42 days	MDA ↓ in muscle, TAC ↑, improves lipid oxidative stability and mitigates oxidative damage	[60]
19	Heat-stressed broilers	*O*. *corniculata* powder + Chromium picolinate (CrPic)	10 g *O. corniculata* powder + 0.2 mg CrPic/kg diet, 1–42 days.	Improvement of microbiota balance, enhancing gut health in heat-stressed broilers, with more significant effects in the early growth stage	[44]
20	Heat-stressed broilers	*O*. *corniculata* powder + CrPic	10 g *O. corniculata* powder + 0.2 mg CrPic/kg diet, 28–42 days	Improves meat quality (crude protein ↑, fat in breast meat ↓) and enhances antioxidant defense against heat stress.	[3]

### 5.2. Anti-Inflammatory Activity

Inflammation is an essential host defense mechanism, but chronic or excessive responses contribute to the pathogenesis of cancer, cardiovascular disorders, and diabetes [68,69]. Recent studies indicate that *O. corniculata* exhibits anti-inflammatory activity through multiple pathways involving its diverse phytochemicals, particularly flavonoids and phenolic acids [70]. These compounds act primarily by inhibiting nuclear factor kappa B (NF-κB)-mediated transcriptional activation, downregulating pro-inflammatory cytokines and enzymes such as cyclooxygenase-2 (COX-2), inducible nitric oxide synthase (iNOS), and phospholipase A_2_ (PLA_2_), and modulating oxidative stress-associated immune responses (Table 3).

In vitro, hot water extracts enriched in 3-O-caffeoylquinic acid and ellagic acid suppressed LPS-induced nitric oxide (NO) production in RAW264.7 cells [62], accompanied by downregulation of *iNOS* and *COX-2* mRNA expression, and suppressed the protein levels of Interleukin-6 (IL-6) and tumor necrosis factor (TNF-α) [62]. Ethanol extracts inhibited NF-κB activation in PC-3 cells by downregulating phosphorylated p65 and IκBα, while upregulating IκBα expression [71]. In vivo, ethanol extracts significantly alleviated colonic inflammation in a rat model of acetic acid-induced inflammatory bowel disease (IBD) [72]. In a LPS-induced acute lung injury (ALI) rat model, the extracts also protected against lung damage by reducing inflammatory cell infiltration and tissue injury [73]. Serum metabolomics further identified flavonoids such as vicenin-3, isovitexin, and isoschaftoside as major absorbed constituents, which were proposed to act via suppression of IL-17/NF-κB signaling and concomitant antioxidant effects [73].

Among isolated compounds, diosmin demonstrated specific anti-inflammatory effects by inhibiting PLA_2_ activity [29]. Evidence from experimental studies suggests that this compound can attenuate venom-induced inflammation and protect against associated tissue injury, further supporting the role of *O. corniculata* flavonoids in modulating inflammatory pathways [29].

**Table 3:** Summary of studies on anti-inflammatory, antipyretic and analgesic activity of *O. corniculata*.

No.	Testing Subjects	Application Part or Compounds	Doses/Duration	Effects	Ref.
1	RAW264.7 cells (LPS 2 μg/mL)	Hot water extract	100–400 μg/mL, 24 h	NO ↓, iNOS ↓, COX-2 ↓, IL-6 ↓, TNF-α ↓	[62]
2	RAW264.7 cells (LPS 0.25 μg/mL)	Ethanol extract	10–50 μg/mL, 24 h	NO ↓, IL-17 ↓; NF-κB pathway ↓	[73]
3	Human RBCs	Diosmin (isolated from *O. corniculata*)	15–120 μM, 2 h	PLA_2_ inhibition ↑, anti-hemolysis, inflammation mediators ↓	[29]
4	Human PC-3 prostate cancer cells	Ethanol extract	50–200 μg/mL, 24 h	Cell proliferation ↓, migration, invasion; apoptosis ↑; NF-κB pathway activity ↓ (p-p65 ↓, p-IκBα ↓, IκBα ↑); anti-inflammatory mechanism via NF-κB inhibition	[71]
5	Swiss albino mice	Diosmin (for snake venom toxicity)	Diosmin (1:200 *w*/*w*) pre-incubated for 1 h (i.p. injection), observation for 5 h	Myotoxicity ↓, pulmonary hemorrhage ↓, CPK ↓, LDH ↓, tissue damage ↓	[29]
6	Mice (acute peritonitis model)	Ethanol extract	6% and 12% extract, p.o., 5 days	Abdominal writhing response ↓ (analgesic); abdominal capillary permeability ↓ (anti-inflammatory)	[74]
7	Swiss mice/Wistar rats	β-sitosterol (extract from leaves)	5–20 mg/kg, i.p., single dose	Analgesia (hot plate latency ↑, writhing ↓), anti-inflammation (paw edema ↓); opioid receptor involvement, PGE inhibition	[70]
8	Rats (acetic acid-induced IBD)	Ethanol extract	200–400 mg/kg, p.o., 7 days	Colon weight ↓, visible lesion score ↓, histopathological score ↓	[72]
9	SD rats (LPS-induced ALI)	Ethanol extract	0.8–3.2 g/kg, p.o., bid × 7 d	Lung edema ↓, inflammatory cell infiltration ↓; serum TNF-α ↓, IL-6 ↓, IL-1β ↓, IL-18 ↓	[73]
10	Rats (pylorus ligation model, indomethacin-induced ulcer model)	Methanol extract	125–500 mg/kg, p.o., single dose	Gastric secretion ↓, acidity ↓, ulcer index ↓; protection against NSAID-induced gastric injury; anti-inflammatory and gastroprotective effects	[75]
11	Rats (CNP model)	Compound prescription containing *O. corniculata*	1 g/kg, p.o., 28 days	Inflammatory infiltration ↓, MCP-1 ↓, ROS ↓, GSH ↑, 4-HNE ↓, ALDH2 ↓, FGF2 ↓; improved tissue pathology;	[48]
12	Rats (CNP model)	Compound prescription containing *O. corniculata*	9.37 g/kg, p.o., 49 days	Prostate index ↓, TNF-α ↓, IL-1β ↓, IL-6 ↓; improved histopathology; cGAS ↓, STING ↓, TRAF6 ↓, HSP70 ↑	[76]

### 5.3. Antibacterial Activity

*O. corniculata* extracts exhibit broad-spectrum antibacterial activity. Methanol extracts containing compounds such as rutin, p-hydroxybenzoic acid, and ferulic acid have been reported to contribute to both antimicrobial and antioxidant effects [61]. Recent findings suggest that the antibacterial effects of *O. corniculata* are associated with the inhibition of biofilm formation and interference with bacterial adhesion and colonization (Table 4) [77].

In vitro, ethanol and aqueous extracts inhibited the growth of both Gram-positive and Gram-negative bacteria, including *S. aureus* and *E. coli*, and were shown to suppress biofilm formation [77,78]. In vivo, methanol extracts reduced intestinal colonization by pathogenic *Shigella* strains in a suckling mouse diarrhea model, with stronger inhibition observed against *S. dysenteriae* type 1 compared to *S. flexneri* 2a [79]. High-performance liquid chromatography (HPLC) analysis suggested that polyphenols are the principal active constituents. The study further showed that efficacy depended on timing of administration, suggesting that the extract may interfere with bacterial adhesion or early colonization processes.

Recent advances in nanotechnology have expanded the antibacterial applications of *O. corniculata* (Table 5). Extracts have been utilized for the eco-friendly synthesis of silver nanoparticles (AgNPs), which display stronger and broader antibacterial activity compared with crude extracts [80]. Hybrid nanomaterials, including silver nanoparticles on graphene oxide (AgNPs@GO) [81] and AgNP-graphene nanocomposites, provide enhanced efficacy by synergistically disrupting bacterial membranes, inhibiting biofilm formation, and interfering with bacterial metabolism [82]. Furthermore, ZnO nanoflowers (ZnO NFs) synthesized using *O. corniculata* extracts damage bacterial membranes and intensify oxidative stress [83]. Flavonoids such as quercetin contribute both antibacterial effects and electron-donating capacity, thereby enhancing the overall activity of these nanostructures [83].

**Table 4:** Summary of studies on the antimicrobial activity of *O. corniculata.*

No.	Testing Subjects	Application Part or Compounds	Methods	Effects	Ref.
1	*S. aureus*, *E. coli*	Aqueous extract	Disk diffusion	At 20% concentration, inhibition zones: *S. aureus* (21 mm), *E. coli* (19.33 mm)	[78]
2	*E. coli*, *S. Typhi*, MDR *S. Typhi*, *K. pneumoniae*, MDR *C. koseri*	Methanol extract	Agar well diffusion, MIC	At 50 mg/mL concentration, inhibition zones: *E. coli* (17 mm), *S. Typhi* (13 mm), MDR *S. Typhi* (16 mm), *K. pneumoniae* (11 mm), *C. koseri* (12 mm). MIC for *E. coli*, *K. pneumoniae*, *C. koseri*: 25 mg/mL; *S. Typhi*: 100 mg/mL; MDR *S. Typhi*: 50 mg/mL.	[84]
3	*S. aureus*	Ethanol extract	Microdilution, biofilm inhibition and eradication assays	At 1% (w/v), antibacterial activity against *S. aureus* was 76.23%; biofilm inhibition rate 71.32% (mid-phase), 69.33% (mature-phase); biofilm eradication rate 64.1%.	[77]
4	*E. coli*, *S. dysenteriae*, *S. typhi*, *B. subtilis*	5-hydroxy-6,7,8,4′-tetramethoxyflavone and 5,7,4′-trihydroxy-6,8-dimethoxyflavone	Agar diffusion assay	The inhibition zones of these two flavonoids isolated from *O. corniculata* ranged from 10 to 16.5 mm.	[85]
5	Suckling mice infected with *S. dysenteriae* 1 (NT4907) and *S. flexneri* 2a (2457T)	Methanol extract	20 mg/kg, single oral administration (simultaneous or 3 h post-infection)	Reduced intestinal colonization of Shigella strains.	[79]
6	Pomfret fish (food preservation model)	Methanol leaf extract (rutin, p-hydroxybenzoic acid, ferulic acid)	Food storage assay (bacterial growth and oxidative stability)	Significantly inhibited *S. aureus* growth during 10 °C storage (48 h) and reduced lipid oxidation, suggesting potential as a natural preservative.	[61]

**Table 5:** Antimicrobial activity of nanomaterials synthesized using *O. corniculata.*

No.	Nanomaterial Type	Biosynthesis Agent	Test Microorganisms	Methods	Effects	Ref.
1	Silver nanoparticles (AgNPs)	*O. corniculata* extract + AgNO_3_	*S. aureus, E. coli, B. subtilis*, *K. pneumoniae*, *S. typhi*, *S. pyogenes*, *P. aeruginosa*, *B. cereus*, *S. typhimurium*, *E. faecalis*, *A. baumannii*, *P. mirabilis*	Disk diffusion, broth microdilution, biofilm inhibition assay	Inhibition zones 10–20 mm (25–50 μg/mL); MIC 0.11–11.5 μg/mL; disrupts membrane integrity, inhibits biofilm, induces ROS, interferes with bacterial metabolic pathways and interacts with intracellular targets	[31,80,86,87,88]
2	AgNPs@GO nanocomposite	*O. corniculata* extract + AgNO_3_ + GO	*B. subtilis*, *E. coli*	Agar well diffusion	Inhibition zones: *B. subtilis* 27 mm, *E. coli* 21 mm. Enhanced antibacterial activity due to synergistic interaction.	[81]
3	AgNPs-graphene nanocomposite	*O. corniculata* extract + AgNO_3_ + graphene	*E. coli*, *S. aureus*, *B. cereus*, *S. typhimurium*	MIC, MBC, inhibition zone	MIC and MBC both as low as 10 μg/mL for AgNPs-graphene composites; disrupts membranes and inhibits metabolism.	[82]
4	ZnO nanoflowers (ZnO NFs)	*O. corniculata* extract + Zn(NO_3_)_2_	*S. aureus*, *E. faecium*, *P. aeruginosa*	Broth dilution	Dose-dependent antibacterial activity (40–120 μg/mL); Gram-positive bacteria more sensitive; induces membrane damage, ROS, and synergizes with flavonoids.	[83]

### 5.4. Anticancer Activity

Research on the anticancer potential of *O. corniculata* has gained momentum in recent years, with investigations employing cell-based, animal, and molecular docking approaches to elucidate its activity across diverse cancer models (Table 6) [63]. Ethanol extracts have been shown to induce apoptosis in MCF-7 breast cancer cells by promoting oxidative stress and modulating apoptosis-related genes, including tumor protein p53 (p53), Fas cell surface death receptor (CD95), and B-cell lymphoma 2 (Bcl-2) [89]. Importantly, these extracts displayed low cytotoxicity toward normal cells, suggesting selective activity. Ethanol and ethyl acetate extracts also exhibited significant cytotoxicity against HepG2 cells, with IC_50_ values of 34.49 μg/mL and 30.25 μg/mL, respectively [63]. Molecular docking identified apigenin, a flavonoid constituent, as a potential inhibitor of epidermal growth factor receptor tyrosine kinase (EGFR-TK), with a binding energy of −7.90 kcal/mol, implicating interference with EGFR signaling [90]. In addition, corniculin, a newly identified lignan from *O. corniculata*, exhibited moderate cytotoxicity against SMMC-7721, MCF-7, HCT-15, and A549 cells, with potency comparable to cisplatin. In vivo, ethanol extracts inhibited tumor progression and prolonged survival in Ehrlich ascites carcinoma (EAC) mice, again without significant toxicity to normal tissues.

Advances in nanotechnology and drug delivery systems have further expanded its anticancer potential. Green-synthesized silver nanoparticles (O-AgNPs) using O. *corniculata* extract exhibited cytotoxicity in MCF-7 and AGS (human gastric carcinoma) cells by inducing apoptosis, while showing low toxicity toward normal cells [88]. Moreover, exosome-mediated delivery of *O. corniculata* polyphenols improved cellular uptake and cytotoxicity in HuH7 hepatocellular carcinoma cells [11]. This strategy promoted apoptosis by upregulating pro-apoptotic genes, including Bcl-2-associated X protein (*Bax*) and *caspase-3*, while downregulating the anti-apoptotic gene *Bcl-2*, thereby enhancing therapeutic efficacy and selectivity.

Antioxidant and anti-inflammatory properties of *O. corniculata* contribute to reducing the risk of cancer development. Selective induction of apoptosis in cancer cells is another important mechanism of its anticancer activity. Evidence suggests that the bioactive constituents of *O. corniculata* possess potential anticancer activity, and further elucidation of mitochondrial signaling, autophagy, and immune modulation may provide valuable insights into its underlying mechanisms.

**Table 6:** Summary of studies on anticancer activity of *O. corniculata*.

No.	Testing Subjects	Application Part or Compounds	Doses/Duration	Effects	Ref.
1	MCF-7 cell line	Ethanol extract	31.25–2000 μg/mL, 24 and 72 h	*p53* ↑, *CD95* ↑, *Bcl-2* ↓; selective cytotoxicity against cancer cells	[89]
2	HepG2 cell line	Ethanol extract and ethyl acetate fraction	35–45 μg/mL, 48 h	Inhibited proliferation of HepG2 cells	[63]
3	SMMC-7721, MCF-7, HCT-15, A549 cancer cell lines	Corniculin	concentration range 0–100 μM, 48 h	Moderate cytotoxicity against multiple cell lines: IC_50_ for SMMC-7721, MCF-7, HCT-15, and A549 cells were 29.0, 35.6, 31.3, and 25.7 μM, respectively	[90]
4	EAC mice	Ethanol extract	100 mg/kg and 400 mg/kg, p.o., 9 days	Inhibited tumor growth; prolonged survival; improved hematological and biochemical parameters; CAT ↑, GSH ↑, MDA ↓; no significant toxicity to normal cells	[91]
5	MCF-7 and AGS cell lines	O-AgNPs	0.1–50 μg/mL, 24 h	Significant cytotoxicity and growth inhibition; induced apoptosis; low toxicity to normal cells	[88]
6	HuH7 cell line	Polyphenols from *O. corniculata* loaded in exosomes	10–100 μg/mL, 24 h	Exosome delivery enhanced cytotoxicity and cellular uptake; promoted apoptosis via upregulation of *Bax* and *caspase-3*, downregulation of *Bcl-2*	[11]

### 5.5. Neuroprotective Activity

Neurodegenerative diseases, including Alzheimer’s disease, Parkinson’s disease, and depression, are commonly associated with oxidative stress and neuroinflammation. These processes drive protein aggregation, neuronal injury, and neurotransmitter imbalance, ultimately resulting in cognitive decline and motor dysfunction [92].

In an AlCl_3_-induced rat model of AD, methanol extracts of *O. corniculata* activated the Nrf2/HO-1 antioxidant pathway, elevated total antioxidant capacity (TAC) and SOD activity, and decreased MDA levels [57]. The extracts restored neurotransmitter balance by increasing dopamine (DA), norepinephrine (NE), and 5-hydroxytryptamine (5-HT), while inhibiting acetylcholinesterase (AChE). They also suppressed neuroinflammation by downregulating TLR4/NF-κB/NLRP3 signaling and reducing pro-inflammatory cytokines. Furthermore, the extracts attenuated endoplasmic reticulum stress by inhibiting PERK/CHOP-mediated apoptosis, upregulated anti-apoptotic *Bcl-2* and autophagy-related *Beclin-1*, and activated Wnt3a/β-catenin signaling. These changes decreased *GSK-3β*, ApoE4, and AD-associated proteins (APP, Aβ, p-Tau), while enhancing low-density lipoprotein receptor-related protein 1 (LRP1)-mediated Aβ clearance. Phytochemical profiling indicated that the extract is rich in flavonoids, organic acids, terpenoids. Behavioral tests demonstrated that the extract significantly improved spatial learning and memory in AD rats, partly due to increased brain-derived neurotrophic factor (BDNF) levels [57].

*O. corniculata* extracts exhibit notable neuroprotective potential by mitigating oxidative stress, neuroinflammation, and neuronal apoptosis, while modulating autophagy and key intracellular signaling cascades. Evidence from multiple neurodegenerative models (Table 7) indicates that these effects are primarily mediated through activation of the Nrf2/HO-1 antioxidant pathway [93,94], inhibition of TLR4/NF-κB/NLRP3-driven inflammatory signaling, and regulation of apoptosis- and autophagy-associated proteins. Collectively, these integrated mechanisms contribute to the alleviation of oxidative stress, preservation of neuronal structure, and improvement of cognitive and motor functions.

**Table 7:** Summary of neuroprotective studies of *O. corniculata.*

No.	Disease Model	Animal Subjects	Application Part or Compounds	Doses/Duration	Effects	Ref.
1	Parkinson’s disease (MPTP-induced)	C57 black male mice	Ethanol extract	250, 500 mg/kg, p.o., co-administered with MPTP	Restored SOD, CAT, reduced LPO; attenuated oxidative stress; improved locomotor activity, muscle coordination, and cognitive performance	[93,94]
2	Parkinson’s disease (Rotenone-induced)	Swiss albino mice	Ethanol extract	500 mg/kg, p.o., for 21 days	Improved motor performance; SOD ↑, CAT ↑, GSH ↑, DA ↑; MDA ↓, NO ↓, Glu ↓; reduced neuronal loss and neuroinflammation	[95]
3	Dementia	Albino mice	Methanol extract	100, 200 mg/kg, p.o., for 21 days	Significantly improved learning and memory abilities	[96]
4	Epilepsy (MES/PTZ-induced)	Wistar rats	Methanol extract	200, 400 mg/kg, i.p., single dose	Restored antioxidant enzymes (SOD, GPx, GR, CAT), reduced LPO; increased DA, NA, 5-HT, GABA; delayed seizures and reduced severity	[97]
5	Alzheimer’s disease (AlCl_3_-induced)	Male SD rats	Methanol extract	150 mg/kg, p.o., weeks	Improved spatial learning and memory; TAC ↑, SOD ↑, MDA ↓; restored DA, NE, 5-HT levels, AChE ↓; upregulation of Nrf2/HO-1, *Bcl-2*, *Beclin-1*, Wnt3a, *β-catenin*, LRP1; downregulation of TLR4/NF-κB/NLRP3, PERK/CHOP, *GSK-3β*, ApoE4; reduced APP, BACE1, Aβ, p-Tau; alleviated neuronal damage and brain pathology.	[57]

## 6. Safety Studies on *O. corniculata*

*O. corniculata* has a long history of medicinal use and is generally considered to have a good safety record. In modern clinical practice, several formulations derived from *O. corniculata* have received approval from the China National Medical Products Administration (NMPA). These include the prescription formulations Gu Kang Capsules, Mi Lin Granules, and Fu Yan Xiao Capsules, all of which are widely used in clinical practice, and no major adverse effects have been reported to date.

Evidence from experimental models further supports the potentially safety profile of *O. corniculata*. A brine shrimp lethality assay indicated low cytotoxicity, with an LC_50_ of 156 µg/mL [52]. In Swiss albino mice [75], oral administration of *O. corniculata* extract at doses up to 2000 mg/kg caused no observable behavioral abnormalities or toxic effects. Li et al. [12] reported that daily oral administration of 0.432 g/kg of crude herb in rats for seven consecutive days caused no observable toxic effects. In a rabbit fracture model, daily dosing of 0.224 g/kg for 30 days produced no adverse reactions [98].

Despite these findings, most studies to date have focused on acute or short-term exposure. Comprehensive data on long-term toxicity, genotoxicity, and reproductive safety remain limited. Moreover, because *O. corniculata* contains calcium oxalate [20], excessive or prolonged intake may pose a theoretical risk of oxalate accumulation, particularly in individuals susceptible to kidney stones [99]. Modern extraction and processing techniques may help to mitigate this risk, but further systematic and quantitative evaluation is necessary to confirm its safety under long-term or high-dose exposure conditions. Preliminary serum metabolomics studies have detected absorbed bioactive constituents after oral administration of *O. corniculata* extract, including flavonoids (e.g., vicenin-3, isovitexin, isoschaftoside) and phenolic acids (e.g., 3-O-, 4-O-, 5-O-p-coumaroylquinic acid) [73]. However, the interactions between different bioactive constituents and their potential effects when combined with other medications have not been systematically evaluated. Further investigation of toxicokinetics, tissue distribution, and maximum tolerated dose (MTD) would be meaningful for confirming safe dosage ranges and supporting its clinical applications.

## 7. Conclusions and Future Prospective

*O. corniculata* exhibits antioxidant, antimicrobial, and anti-inflammatory activities, and its wide distribution, ease of cultivation, and chemical diversity underscore its potential as a sustainable bioresource. Flavonoids are the principal absorbed constituents in vivo and likely mediate antioxidant and anti-inflammatory effects through multiple signaling pathways [73]. Recent studies have also identified structurally unique compounds, such as the alkaloid ATA, which represents a promising lead for α-glucosidase inhibition [39], and the lignan corniculin A, which demonstrates moderate anticancer activity comparable to cisplatin [100]. Advances in nanotechnology have further expanded its therapeutic potential. Green-synthesized nanomaterials derived from *O. corniculata* extracts exhibit enhanced antioxidants, antibacterial and anticancer activities. Exosome-mediated delivery systems for polyphenols also improve bioavailability, addressing limitations of conventional herbal extracts [11]. Collectively, these findings highlight the broad application potential of *O. corniculata*, as outlined below:

(1) Dietary supplements and nutraceuticals: Flavonoids and polysaccharides enhance antioxidant defenses and help mitigate disorders associated with oxidative stress, supporting their potential development as dietary supplements and nutraceuticals [7].
(2) Antimicrobial applications: Extracts exhibit antibacterial activity against *S. aureus* and *E. coli*, with topical formulations such as antibacterial creams showing efficacy [78]. Green-synthesized nanomaterials further enhance antibacterial activity, offering opportunities for wound-care and infection-control products.
(3) Hypoglycemic potential: Ethanol extracts exhibit antihyperglycemic effects in STZ-induced diabetic rats, mainly through the enhancement of antioxidant defenses and protection of pancreatic β-cells [8]. The alkaloid ATA has been identified as one of the contributors to this activity [37,39]. These findings suggest that the plant’s bioactive constituents hold promise for the development of natural hypoglycemic agents.
(4) Food preservation: Polyphenols delay lipid peroxidation in animal fats and extend shelf life. Oil-soluble extracts show stronger antioxidant effects than water-soluble forms, with optimal activity at 0.02–0.04% concentrations, comparable to butylated hydroxyanisole (BHA) and butylated hydroxytoluene (BHT) [77]. Methanol extracts of *O. corniculata* effectively inhibit bacterial growth and oxidative spoilage in fish meat, indicating its potential as a natural preservative [61].
(5) Poultry production: Dietary supplementation with *O. corniculata* improves antioxidant stability of broiler meat and increases the levels of bioactive compounds such as lutein, zeaxanthin, and PUFAs [4]. It also optimizes the fatty acid profile, helps maintain gut microbiota balance, alleviates heat stress, and supports growth performance [44,60].

Future research should prioritize standardized cultivation and harvesting practices, as geographic origin, environmental conditions, and plant varieties strongly influence the accumulation and composition of bioactive compounds. Standardized extraction and quality-control protocols, particularly those based on antioxidant-rich components such as flavonoids and polyphenols, will be essential to ensure reproducibility. Mechanistic studies are also needed to clarify how these constituents exert their biological effects and to guide practical applications. Systematic evaluation of pharmacokinetics and long-term safety is also required to support clinical translation.

Considering its multi-target pharmacological properties, *O. corniculata* may have potential for synergistic or modulatory effects when combined with antibacterial, anticancer, or neuroprotective drugs. However, such interactions have not yet been systematically investigated and warrant further study. Moreover, mechanistic investigations focusing on the antioxidant, anti-inflammatory, and gut microbiota-modulating effects of *O. corniculata* in poultry production will be valuable to clarify its roles and optimize its practical application as a feed additive. With its low cost, broad availability, and multi-target bioactivities, *O. corniculata* holds considerable promise as a sustainable botanical resource for improving animal health, food preservation, and the development of novel antioxidant-based therapeutics.

## Figures and Tables

**Table 1:** Traditional uses of *O. corniculata.*

Country	Components and Preparation Methods	Applications	Prescription Source
China	Harvested in April and May, shade-dried, and pounded for juice.	Treats sores; pounded and applied to kill parasites.	Xin Xiu Ben Cao (Tang Dynasty, 659 AD) [45]
China	Pounded for juice and prepared with vinegar	Indicated for strangury, hematuria, dysuria, and pediatric gingival ulceration with bleeding and fetor.	Zheng Lei Ben Cao (Song Dynasty, 1082 AD) [45]
China	Pounded for juice and prepared with wine	Treats strangury with painful reddish urination and bladder heat; for postpartum uterine prolapse.	Shi Yi De Xiao Fang (Yuan Dynasty, 1337 AD) [45]
China	Decocted with Bassia scoparia seeds and Plantago asiatica seeds	Treats strangury and leukorrhea.	Ben Cao Gang Mu (Ming Dynasty, 1578 AD) [49]
China	Leaves harvested in summer and shade-dried. Juice prepared with wine, honey, or sugar.	For sores, thirst, strangury, urinary retention, and difficult labor; for red and white vaginal discharge in women.	Zheng Zhi Zhun Sheng (Ming Dynasty, 1602 AD) [45]
China	Decocted with rock sugar and taken orally	For chronic diarrhea and various types of dysentery.	Dian Nan Ben Cao (Ming Dynasty, 1436 AD) [49]
China	Pounded for juice and prepared with wine, vinegar, honey, or sugar	For strangury, carbuncles, heat toxins, burns, and venomous bites; promotes urination and defecation. for phlegm disorders and anal swelling.	De Pei Ben Cao (Qing Dynasty, 1761 AD) [45]
India	With turmeric and Emblica officinalis in decoction; leaf juice with honey as mouth rinse; also consumed and used as fodder.	For diarrhea, oral ulcers, skin infections, and lowering blood glucose.	Ayurvedic Tradition [6,50,51]
Tanzania	Whole plant decoction; crushed fresh leaves applied topically	Treatment of skin infections, wounds, conjunctivitis, sore throat, fever, gastrointestinal discomfort	Ethnobotanical practice in Tanzania [52]
/	Used fresh, juiced, made into paste or decoction; administered orally or topically	Treat liver diseases, jaundice, skin disorders, and urinary tract infections	Traditional medical systems: Ayurveda, Unani, and Siddha [53]
Pakistan	Fresh leaves boiled in water for 2 h and placed for cooling.	For vitamin C deficiency and mouth smell	Traditional Persian Medicine [54]
China	Combined with Musa basjoo rhizome, Psoralea corylifolia, Dipsacus asper, etc	For fractures, coxarthritis, and osteoporosis associated with liver-kidney deficiency and meridian obstruction.	Gu Kang Capsule (www.nmpa.gov.cn)
China	Combined with Alternanthera philoxeroides, Agrimonia pilosa, Plantago asiatica, etc	For urinary tract infections, including cystitis, strangury, dysuria, and painful urination.	Mi Lin Qing Capsule (www.nmpa.gov.cn/)
China	Combined with Boenninghausenia albiflora, Polygonum capitatum and Plantago asiatica	For treating stranguria caused by damp-heat accumulation, characterized by difficult urination, dribbling, and painful urination.	Mi Ling Granule/Capsule (www.nmpa.gov.cn/)
China	Combined with Patrinia scabiosaefolia, Rheum palmatum, Paeonia suffruticosa bark, ect	Adjuvant treatment for chronic gynecological inflammation; also regulates menstruation and relieves pain.	Fu Yan Xiao Capsule (www.nmpa.gov.cn/)
China	Combined with Root of Musa basjoo and Curcuma longa	For traumatic injuries, contusions, and soft tissue damage with swelling and pain.	Zhong Tong Shu Spray (www.nmpa.gov.cn/)

## Data Availability

The original contributions presented in the study are included in the article; further inquiries can be directed to the corresponding author.

## Supplementary Materials

The following supporting information can be downloaded at: https://www.mdpi.com/article/10.3390/antiox14111352/s1. Figure S1: Structures of flavones (1–48) isolated from *O. corniculata*. Figure S2: Structures of other flavonoid subclasses isolated from *O. corniculata*, including flavonols (49–67), dihydroflavones (68–70), isoflavones (71–79), chalcone (80), dihydrochalcones (81–82), and flavanols (83–84). Figure S3: Structures of organic acids identified from *O. corniculata*. (A): Phenolic acids (85–107); (B): Other organic acids (108–135). Figure S4: Structures of terpenoids (136–155) identified from *O. corniculata*. Figure S5: Structures of alkaloids identified from *O. corniculata*. Table S1: Chemical compounds isolated from *O. corniculata*. References [19,20,21,22,23,27,29,31,35,37,39,42,57,61,62,63,68,70,83,85,90,100,101,102,103,104,105,106,107,108,109] are cited in the supplementary materials.
