# Supplementary material for: Oxalis corniculata L. As a Source of Natural Antioxidants: Phytochemistry, Bioactivities, and Application Potential
Source: Antioxidants (Basel). 2025 Nov 11;14(11):1352. doi: 10.3390/antiox14111352 (PMC12649735; doi:10.3390/antiox14111352)
Supplement: Supplementary file 1 [file antioxidants-14-01352-s001.zip › antioxidants-3910290-supplementary.pdf]

Supplementary Materials

Oxalis corniculata L. as a Source of Natural Antioxidants: Phytochemistry, Bioactivities, and Application Potential

Tao Zhong [1,2], Junying He [1,2], Hao Zhao [1,2], Chang Tan [1,2], Wenjing Zhou [1,2] Congming Wu [1,2] and Jijun Kang [1,2,*]

1 Technology Innovation Center for Food Safety Surveillance and Detection (Hainan), Sanya Institute of China Agricultural University, Sanya, 572025, China

2 State Key Laboratory of Veterinary Public Health and Safety, College of Veterinary Medicine, China Agricultural University, Beijing 100193, China

* Corresponding author: kangjijun@cau.edu.cn

Table S1. Chemical compounds isolated from *O. corniculata.*

NO.	Compounds	Molecular Formula	Parts	Refs.
Flavonoids				
1	Luteolin	$C_{15}H_{10}O_6$	Whole herb	[1-4]
2	Luteolin-O-rutinoside	$C_{27}H_{30}O_{15}$	Whole herb	[2]
3	Luteolin-3',7-di-O-glucoside	$C_{27}H_{30}O_{16}$	Whole herb	[2]
4	Luteolin-7-O-β-D-glucoside	$C_{21}H_{20}O_{11}$	Whole herb	[3]
5	Lonicerin	$C_{27}H_{30}O_{15}$	Whole herb	[1, 2]
6	Diosmetin	$C_{16}H_{12}O_6$	Whole herb	[1, 2, 5]
7	Diosmetin 7-O-beta-D-glucopyranoside	$C_{22}H_{22}O_{11}$	Leave	[6]
8	Diosmin	$C_{28}H_{32}O_{15}$	Whole herb	[4, 7]
9	Luteolin-6-C-glucose-2″-O-rhamnoside	$C_{27}H_{30}O_{15}$	Whole herb	[2]
10	Apigenin	$C_{15}H_{10}O_5$	Whole herb	[1, 2, 4, 8, 9]
11	Apigenin-6,8-di-C-β-D-glucoside	$C_{27}H_{30}O_{15}$	Whole herb	[2]
12	Apigenin 7,4′-diglucoside	$C_{27}H_{30}O_{15}$	Leave	[6]
13	Isoschaftoside	$C_{26}H_{28}O_{14}$	Whole herb	[1, 2, 10]
14	Neoschaftoside	$C_{26}H_{28}O_{14}$	Whole herb	[10]
15	Cosmosiin	$C_{21}H_{20}O_{10}$	Whole herb	[2]
16	Vitexin	$C_{21}H_{20}O_{10}$	Whole herb	[2, 6]
17	Vitexin -4''-O-glucoside	$C_{27}H_{30}O_{15}$	Whole herb	[1]
18	Vitexin 2''-O-rhamnoside	$C_{27}H_{30}O_{14}$	Whole herb	[1]
19	Vitexin 6''-O-malonyl 2''-O-xyloside	$C_{29}H_{30}O_{17}$	Leave	[11]
20	Isovitexin	$C_{21}H_{20}O_{10}$	Whole herb, Leave	[1, 2, 5, 6, 10, 12]
21	Isovitexin-7-O-glucoside	$C_{27}H_{30}O_{15}$	Whole herb	[2]
22	Isovitexin-2''-O-rhamnoside	$C_{27}H_{30}O_{14}$	Whole herb	[2]
23	Swertisin	$C_{22}H_{22}O_{10}$	Whole herb, Leave	[1, 2, 5, 6]
24	Swertiajaponin	$C_{22}H_{22}O_{11}$	Whole herb	[1]
25	Isoorientin	$C_{21}H_{20}O_{11}$	Whole herb	[1, 2, 10]
26	Spinosin	$C_{28}H_{32}O_{15}$	Whole herb	[1]
27	Jaceosidin	$C_{17}H_{14}O_7$	Whole herb	[1]
28	Hispidulin	$C_{16}H_{12}O_6$	Whole herb	[1]
29	5-Demethylnobiletin	$C_{20}H_{20}O_8$	Whole herb	[13]
30	Sinensetin	$C_{20}H_{20}O_7$	Whole herb	[1]
31	Isosinensetin	$C_{20}H_{20}O_7$	Whole herb	[1]
32	Orientin	$C_{21}H_{20}O_{11}$	Whole herb	[1, 2, 14]
33	Chryseriol	$C_{16}H_{12}O_6$	Whole herb	[1, 4]
34	Scoparin	$C_{22}H_{22}O_{11}$	Whole herb	[1]
35	Chrysin	$C_{15}H_{10}O_4$	Whole herb	[4]
36	Tectochrysin	$C_{16}H_{12}O_4$	Whole herb	[4]
37	Acacetin	$C_{16}H_{12}O_5$	Whole herb	[4, 9]
38	Moslosooflavone	$C_{17}H_{14}O_5$	Whole herb	[13]

39	Tricin-7-O-β-D-glucopyranoside	$C_{23}H_{24}O_{13}$	Whole herb	[1]
40	5-hydroxy-6,7,8,4′-tetramethoxyflavone	$C_{19}H_{18}O_7$	Whole herb	[9, 15]
41	5,7,4′-trihydroxy-6,8-dimethoxyflavone	$C_{17}H_{14}O_7$	Whole herb	[15]
42	Vicenin 2	$C_{27}H_{30}O_{15}$	Whole herb	[1]
43	Vicenin 3	$C_{26}H_{28}O_{14}$	Whole herb	[1]
44	Carlinoside	$C_{26}H_{28}O_{15}$	Whole herb	[1]
45	Tangeretin	$C_{20}H_{20}O_7$	Whole herb	[1]
46	Nobiletin	$C_{21}H_{22}O_8$	Whole herb	[1]
47	N/A	$C_{30}H_{28}O_{13}$	Whole herb	[13]
48	Corniculatin A	$C_{30}H_{28}O_{13}$	Whole herb	[3, 16]
49	Penduletin	$C_{18}H_{16}O_7$	Whole herb	[13]
50	5-Hydroxy-3,6,7,4'-tetramethoxyflavone	$C_{19}H_{18}O_7$	Whole herb	[13]
51	2-(3,4-Dimethoxyphenyl)-3,6,7-trimethoxychromen-4-one	$C_{20}H_{20}O_7$	Whole herb	[13]
52	3,5,2'-Trihydroxy-7,5'-dimethoxyflavone	$C_{17}H_{14}O_7$	Whole herb	[13]
53	Rhamnazin	$C_{17}H_{14}O_7$	Whole herb	[1]
54	Isorhamnetin 3-glucoside-7-rhamnoside	$C_{28}H_{32}O_{16}$	Whole herb	[1]
55	Quercetin	$C_{15}H_{10}O_7$	Whole herb	[1, 2, 4, 17, 18]
56	Quercetin 3-rutinoside-7-glucoside	$C_{33}H_{40}O_{21}$	Whole herb	[1]
57	Quercetin 3-O-glucuronide	$C_{21}H_{18}O_{13}$	Whole herb	[1]
58	Rutin	$C_{27}H_{30}O_{16}$	Leave	[19]
59	Kaempferol	$C_{15}H_{10}O_6$	Whole herb	[1, 17]
60	Kaempferol 3-glucoside 7-rhamnoside	$C_{27}H_{30}O_{15}$	Whole herb	[10]
61	Kaempferol 3-O-sophorotrioside	$C_{33}H_{40}O_{21}$	Leave	[11]
62	Kaempferol 3-O-glucuronide	$C_{21}H_{18}O_{12}$	Leave	[11]
63	kaempferol-3-O-alpha-L-rhamnoside	$C_{21}H_{20}O_{10}$	Whole herb	[14]
64	Kaempferitrin	$C_{27}H_{30}O_{14}$	Aerial part	[20]
65	Panasenoside	$C_{27}H_{30}O_{16}$	Whole herb	[10]
66	Betuletol	$C_{17}H_{14}O_7$	Whole herb	[13]
67	Eupalitin	$C_{17}H_{14}O_7$	Whole herb	[13]
68	Naringenin-7-O-beta-D-glucuronide	$C_{21}H_{20}O_{11}$	Whole herb	[10]
69	Hesperitin	$C_{16}H_{14}O_6$	Whole herb	[4]
70	Rhusflavone	$C_{30}H_{22}O_{10}$	Whole herb	[4]
71	4''-Methyloxy-genistin	$C_{22}H_{22}O_{10}$	Whole herb	[10]
72	Tectoridin	$C_{22}H_{22}O_{11}$	Whole herb	[2]
73	Tectorigenin	$C_{16}H_{12}O_6$	Aerial part	[20]

74	Iristectorigenin B	$C_{17}H_{14}O_7$	Whole herb	[2]
75	iristectorigenin A	$C_{17}H_{14}O_7$	Aerial part	[20]
76	Irigenin	$C_{18}H_{16}O_8$	Aerial part	[20]
77	Daidzin	$C_{21}H_{20}O_9$	Whole herb	[1]
78	Formononetin	$C_{16}H_{12}O_4$	Whole herb	[1]
79	3'-Hydroxy puerarin	$C_{21}H_{20}O_{10}$	Whole herb	[10]
80	Isoliquiritin	$C_{21}H_{22}O_9$	Whole herb	[4]
81	Phlorizin	$C_{21}H_{24}O_{10}$	Whole herb	[4]
82	Naringin Dihydrochalcone	$C_{27}H_{34}O_{14}$	Whole herb	[4]
83	Epicatechin	$C_{15}H_{14}O_6$	Whole herb	[1, 10]
84	Epigallocatechin	$C_{15}H_{14}O_7$	Whole herb	[1]
Organic acids				
85	Protocatechuic acid	$C_7H_6O_4$	Whole herb	[1, 2, 4]
86	Vanillic acid	$C_8H_8O_4$	Leave	[6, 21]
87	Chlorogenic acid	$C_{16}H_{18}O_9$	Whole herb	[2, 4]
88	Cryptochlorogenic acid	$C_{16}H_{18}O_9$	Whole herb	[1]
89	Caffeic acid	$C_9H_8O_4$	Leave	[11]
90	1-Caffeoylquinic acid	$C_{16}H_{18}O_9$	Whole herb	[2]
91	3-O-Caffeoylquinic acid	$C_{16}H_{18}O_9$	Whole herb, Leave	[11, 22]
92	Isochlorogenic acid A	$C_{25}H_{24}O_{12}$	Whole herb	[1]
93	Isochlorogenic acid B	$C_{25}H_{24}O_{12}$	Whole herb	[1]
94	Rosmarinic acid	$C_{18}H_{16}O_8$	Whole herb	[4]
95	Ferulic acid	$C_{10}H_{10}O_4$	Whole herb, Leave	[1, 2, 17, 18]
96	3-O-Feruloylquinic acid	$C_{17}H_{20}O_9$	Whole herb	[1, 2]
97	4-O-Feruloylquinic acid	$C_{17}H_{20}O_9$	Whole herb	[1]
98	Gallic acid	$C_7H_6O_5$	Whole herb	[1, 19, 21]
99	p-Hydroxybenzoic acid	$C_7H_6O_3$	Whole herb, Leave	[4, 6, 9, 11, 17, 18]
100	Eschweilenol C	$C_{20}H_{16}O_{12}$	Leave	[11]
101	Coumaroyl quinic acid	$C_{16}H_{18}O_8$	Whole herb	[4]
102	Gentisic Acid	$C_7H_6O_4$	Whole herb	[1]
103	p-Coumaric acid	$C_9H_8O_3$	Leave	[11]
104	4-O-p-Coumaroylquinic acid	$C_{16}H_{18}O_8$	Whole herb	[1]
105	5-O-p-Coumaroylquinic acid	$C_{16}H_{18}O_8$	Whole herb	[1]
106	Sinapic acid	$C_{11}H_{12}O_5$	Leave	[11]
107	Glucosyringic acid	$C_{15}H_{20}O_{10}$	Whole herb	[1]
108	Cinnamic acid	$C_9H_8O_2$	Whole herb	[4]
109	Aconitic acid	$C_6H_6O_6$	Whole herb	[1]
110	Quinate	$C_7H_{12}O_6$	Whole herb	[1]
111	Azelaic acid	$C_9H_{16}O_4$	Whole herb	[1, 2]
112	Malic acid	$C_4H_6O_5$	Whole herb, Stem	[4, 23]
113	Gluconic acid	$C_6H_{12}O_7$	Whole herb	[4]
114	Mucic acid	$C_6H_9O_8$	Whole herb	[4]
115	Citric acid	$C_6H_8O_7$	Whole herb, Stem	[1, 9, 23]
116	Phenylpyruvic acid	$C_9H_8O_3$	Whole herb	[10]

117	3-Methyladipic acid	$C_7H_{12}O_4$	Whole herb	[10]
118	Palmitic acid	$C_{16}H_{32}O_2$	Whole herb, Leave	[2, 4, 9, 24-26]
119	Linolenic acid	$C_{18}H_{30}O_2$	Whole herb, Leave	[1, 4, 9]
120	α-Eleostearic acid	$C_{18}H_{30}O_2$	Whole herb	[1]
121	Tetradecanoic acid	$C_{14}H_{28}O_2$	Leave	[24, 25]
122	Octadecanoic acid	$C_{18}H_{36}O_2$	Whole herb, Leave	[4, 25]
123	cis-9-Hexadecenoic acid	$C_{16}H_{30}O_2$	Leave	[25]
124	Hexadecenoic acid, Z-11-	$C_{16}H_{30}O_2$	Leave	[25]
125	Oleic Acid	$C_{18}H_{34}O_2$	Leave	[25]
126	Arachidonic acid	$C_{20}H_{32}O_2$	Whole herb, Leave	[4, 25]
127	Eicosanoic acid	$C_{20}H_{40}O_2$	Leave	[25]
128	Linoleic acid	$C_{18}H_{32}O_2$	Whole herb, Leave	[4, 25, 26]
129	6-Octadecenoic acid	$C_{18}H_{34}O_2$	Leave	[25]
130	cis-13-Octadecenoic acid	$C_{18}H_{34}O_2$	Leave	[25]
131	cis-Vaccenic acid	$C_{18}H_{34}O_2$	Leave	[25]
132	17-Hydroxylinolenic acid	$C_{18}H_{30}O_3$	Whole herb	[4]
133	Erucic acid	$C_{22}H_{42}O_2$	Whole herb	[26]
134	Pentadecanoic acid	$C_{15}H_{30}O_2$	Whole herb	[4]
135	2-Hydroxypalmitic acid	$C_{16}H_{32}O_3$	Whole herb	[4]
Terpenoids				
136	β-Pinene	$C_{10}H_{16}$	Leave	[23]
137	Camphene	$C_{10}H_{16}$	Leave	[24]
138	β-Myrcene	$C_{10}H_{16}$	Leave	[24]
139	(E)-Ocimene	$C_{10}H_{16}$	Leave	[24]
140	cis-Carveol	$C_{10}H_{16}O$	Leave	[24]
141	trans-Carveol	$C_{10}H_{16}O$	Leave	[24]
142	Terpinolene	$C_{10}H_{16}$	Leave	[24]
143	Geraniol	$C_{10}H_{18}O$	Leave	[24]
144	Linalool	$C_{10}H_{18}O$	Leave	[24]
145	Linalool oxide	$C_{10}H_{18}O_2$	Leave	[24]
146	Geranyl acetate	$C_{12}H_{20}O_2$	Leave	[24]
147	Terpinen-4-ol	$C_{10}H_{18}O$	Leave	[24]
148	Geranialdehyde	$C_{10}H_{16}O$	Leave	[23]
149	Safranal	$C_{10}H_{14}O$	Leave	[24]
150	trans-Pinocarveol	$C_{10}H_{16}O$	Leave	[23]
151	Verproside	$C_{22}H_{26}O_{13}$	Whole herb	[10]
152	Oleanolic acid	$C_{30}H_{48}O_3$	Whole herb	[4]
153	Eburicoic acid	$C_{31}H_{50}O_3$	Aerial part	[27]
154	Squalene	$C_{30}H_{50}$	Leave	[25, 26]
155	Phytol	$C_{20}H_{40}O$	Leave	[25]
Alkaloids				
156	Trigonelline	$C_7H_7NO_2$	Whole herb	[1, 2, 10]
157	Betaine	$C_5H_{11}NO_2$	Whole herb	[1, 10]
158	Nicotinamide	$C_6H_6N_2O$	Whole herb	[1]

159	Noroxyhydrastinine	$C_{10}H_9NO_3$	Whole herb	[10]
160	Aspergillus triazolate A	$C_{12}H_{21}N_3O_2$	Whole herb	[28]
Lignan				
161	Corniculin	$C_{21}H_{18}O_6$	Whole herb	[29]
Coumarins				
162	Scopolin	$C_{16}H_{18}O_9$	Whole herb	[1]
163	Hymecromone	$C_{10}H_8O_3$	Whole herb	[1]
164	4-Methoxycoumarin	$C_{10}H_8O_3$	Whole herb	[1]
165	Coumarin	$C_9H_6O_2$	Whole herb	[1]
166	Umbelliferone	$C_9H_6O_3$	Whole herb	[1]
167	10-O-Coumaroyl-10-O-deacetylasperuloside	$C_{25}H_{26}O_{12}$	Whole herb	[10]
Simple phenolics				
168	6-Gingerol	$C_{17}H_{26}O_4$	Whole herb	[1, 2]
169	Furanmethanol	$C_5H_6O_2$	Leave	[25]
170	Catechol	$C_6H_6O_2$	Leave	[19, 25]
171	5-Hydroxymethylfurfural	$C_6H_6O_3$	Leave	[25]
172	2-Methoxy-4-vinylphenol	$C_9H_{10}O_2$	Leave	[25]
173	Ellagic Acid	$C_{14}H_6O_8$	Whole herb	[22]
Fatty acid esters				
174	Methyl palmitoleate	$C_{17}H_{32}O_2$	Whole herb	[4]
175	Ethyl linolenate	$C_{20}H_{34}O_2$	Whole herb	[4]
176	Hexadecanoic acid, ethyl ester	$C_{18}H_{36}O_2$	Leave	[25, 26]
177	9,12-Octadecadienoic acid, methyl ester, (E,E)	$C_{19}H_{34}O_2$	Leave	[26]
Anthraquinones				
178	Rhein	$C_{15}H_8O_6$	Whole herb	[5]
179	Ruberythric acid	$C_{25}H_{26}O_{13}$	Whole herb	[10]
180	Emodin-8-o-beta-gentiobioside	$C_{27}H_{30}O_{15}$	Whole herb	[10]
181	Chrysophanol 8-O-glucoside	$C_{21}H_{20}O_9$	Whole herb	[10]
Aldehydes				
182	Protocatechuic aldehyde	$C_7H_6O_3$	Whole herb	[1, 2]
183	Cinnamaldehyde	$C_9H_7O_9$	Whole herb	[4]
184	p-Hydroxybenzaldehyde	$C_7H_6O_2$	Whole herb	[5]
185	9,17-Octadecadienal, (Z)-	$C_{18}H_{32}O$	Leave	[25]
186	2-Isopropylbenzaldehyde	$C_{10}H_{12}O$	Leave	[24]
187	4-Propyl benzaldehyde	$C_{10}H_{12}O$	Leave	[24]
188	2,4-Decadienal	$C_{10}H_{16}O$	Leave	[24]
189	n-Nonanal	$C_9H_{18}O$	Leave	[24]
Glycoside				
190	β-sitosterol-3-O-beta-d-glucoside	$C_{35}H_{60}O_6$	Whole herb	[3]
191	Vanillyl beta - D - glucopyranoside	$C_{14}H_{20}O_8$	Whole herb	[4]
192	Rubrofusarin-6-O-beta-D-gentiobioside	$C_{27}H_{32}O_{15}$	Whole herb	[10]
Sterols				
193	β-Sitosterol	$C_{29}H_{50}O$	Leave	[9, 25, 30]

194	γ-Sitosterol	$C_{29}H_{50}O$	Leave	[25]
195	Campesterol	$C_{28}H_{48}O$	Leave	[25]
Carbohydrates				
196	D(-)-Fructose	$C_6H_{12}O_6$	Whole herb	[5]
197	Sucrose	$C_{12}H_{22}O_{11}$	Whole herb	[1]
Amino acid				
198	Tyrosine	$C_9H_{11}NO_3$	Whole herb	[1]
199	Leucine	$C_6H_{13}NO_2$	Whole herb	[1]
200	Arginine	$C_6H_{14}N_4O_2$	Whole herb	[1]
201	D-Pyroglutamic acid	$C_5H_7NO_3$	Whole herb	[1]
202	L-phenylalanine	$C_9H_{11}NO_2$	Whole herb	[1]
203	D-proline	$C_5H_9NO_2$	Whole herb	[10]
204	D-Leucine	$C_6H_{13}NO_2$	Whole herb	[10]
205	4-Aminobutyric acid	$C_4H_9NO_2$	Whole herb	[10]
Carotenoids and pigments				
206	Phytofluene	$C_{40}H_{62}$	Leave	[11]
207	Pheophorbide b	$C_{35}H_{34}N_4O_6$	Leave	[11]
208	Pheophytin a	$C_{55}H_{74}N_4O_6$	Leave	[11]
209	Pheophytin b	$C_{55}H_{72}N_4O_6$	Leave	[11]
210	All-E-neoxanthin	$C_{40}H_{56}O_4$	Leave	[11]
211	All-E-violaxanthin	$C_{40}H_{56}O_4$	Leave	[11]
212	All-E-lutein	$C_{40}H_{56}O_2$	Leave	[11]
213	Hydroxy-pheophytin a	/	Leave	[11]
214	Hydroxy-pheophytin a'	/	Leave	[11]
215	Hydroxy-pheophytin b	/	Leave	[11]
216	13-Hydroxy-chlorophyll b	/	Leave	[11]
217	Chlorophyll a	$C_{55}H_{72}MgN_4O_5$	Leave	[11]
218	Chlorophyll b	$C_{55}H_{70}MgN_4O_6$	Leave	[11]
219	Chlorophyll b'	$C_{55}H_{70}MgN_4O_6$	Leave	[11]
220	Chlorophyll b epimer	$C_{55}H_{70}MgN_4O_6$	Leave	[11]
Nucleosides and bases				
221	Guanosine	$C_{10}H_{13}N_5O_5$	Whole herb	[5, 10]
222	Adenine	$C_5H_5N_5$	Whole herb	[1, 5, 10]
Vitamins and metabolites				
223	Pantothenic acid	$C_9H_{17}NO_5$	Whole herb	[21]
224	Myo-Inositol	$C_6H_{12}O_6$	Whole herb	[10]
225	Vitamin C	$C_6H_8O_6$	Leave	[19, 23, 31]
226	α-Tocopherol	$C_{29}H_{50}O_2$	Leave	[19]
227	DL-alpha-Tocopherol	$C_{29}H_{50}O_2$	Leave	[25]

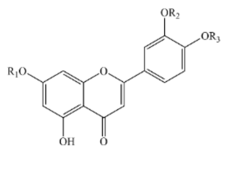

1 R_1=H, R_2=H, R_3=H
2 R_1=α-L-Rha-(1→6)-β-D-Glc, R_2=H, R_3=H
3 R_1=β-D-Glc, R_2=β-D-Glc, R_3=H
4 R_1=β-D-Glc, R_2=H, R_3=H
5 R_1=α-L-Rha-(1→2)-β-D-Glc, R_2=H, R_3=H
6 R_1=H, R_2=H, R_3=CH_3
7 R_1=β-D-Glc, R_2=H, R_3=CH_3
8 R_1=α-L-Rha-(1→6)-β-D-Glc, R_2=H, R_3=CH_3

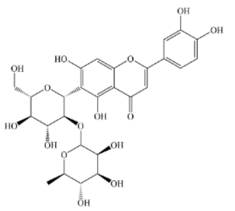

9

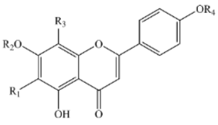

10 R_1=H, R_2=H, R_3=H, R4=H
11 R_1=β-D-Glc, R_2=H, R_3=β-D-Glc, R4=H
12 R_1=H, R_2= β-D-Glc, R_3=H, R4= β-D-Glc
13 R_1=α-L-Ara, R_2=H, R_3=β-D-Glc, R4=H
14 R_1=β-D-Glc, R_2=H, R_3=α-L-Ara, R4=H
15 R_1=H, R_2=β-D-Glc, R_3=H, R4=H

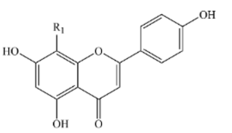

16 R_1=β-D-Glc
17 R_1=α-L-Rha-(1→2)-β-D-Glc
18 R_1=β-D-Glc-(1→4)-β-D-Glc

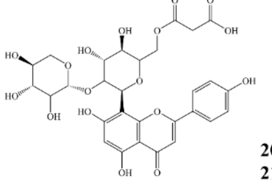

19

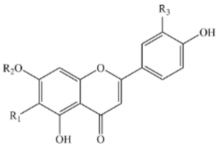

20 R_1=β-D-Glc, R_2=H, R_3=H
21 R_1=β-D-Glc, R_2= β-D-Glc, R_3=H
22 R_1=α-L-Rha-(1→2)-β-D-Glc, R_2=H, R_3=H
23 R_1=β-D-Glc, R_2=CH_3, R_3=H
24 R_1=β-D-Glc, R_2=CH_3, R_3=OH
25 R_1=β-D-Glc, R_2=H, R_3=OH
26 R_1=β-D-Glc-(1→2)-β-D-Glc, R_2= CH_3, R_3=H
27 R_1=OCH_3, R_2= H, R_3=OCH_3
28 R_1=OCH_3, R_2= H, R_3=H

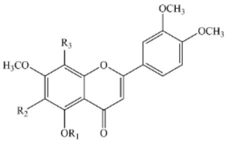

29 R_1=H, R_2=OCH_3, R_3=OCH_3
30 R_1=CH_3, R_2=OCH_3, R_3=H
31 R_1=CH_3, R_2=H, R_3=OCH_3

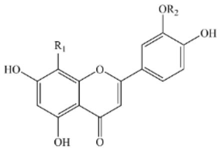

32 R_1=β-D-Glc, R_2=H
33 R_1=H, R_2=CH_3
34 R_1=β-D-Glc, R_2=CH_3

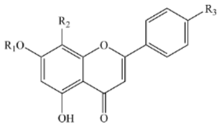

35 R_1=H, R_2=H, R_3=H
36 R_1=CH_3, R_2=H, R_3=H
37 R_1=H, R_2=H, R_3=OCH_3
38 R_1=CH_3, R_2=OCH_3, R_3=H

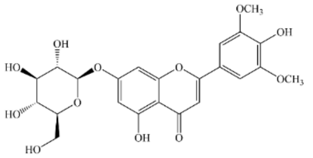

39

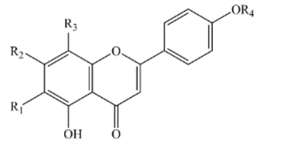

40 R_1=OCH_3, R_2=OCH_3, R_3=OCH_3, R_4=OCH_3
41 R_1=OCH_3, R_2=OH, R_3=OCH_3, R_4=OH
42 R_1=β-D-Glc, R_2=OH, R_3=β-D-Glc, R_4=OH
43 R_1=β-D-Glc, R_2=OH, R_3=β-D-Xyl, R_4=OH

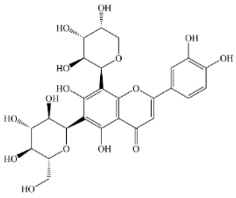

44

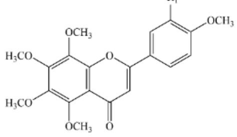

45 R_1=H
46 R_1=OCH_3

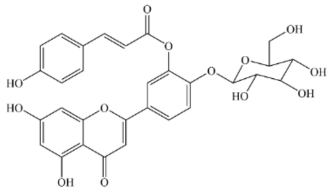

47

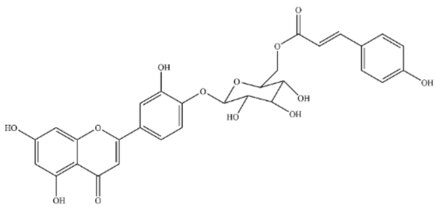

48

Supplemental Figure S1. Structures of flavones (1–48) isolated from *O. corniculata*.

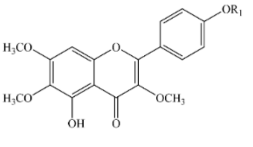

49 R_1=H
50 R_1=CH_3

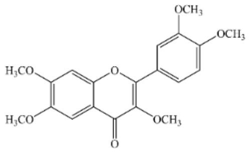

51

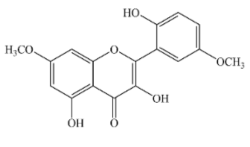

52

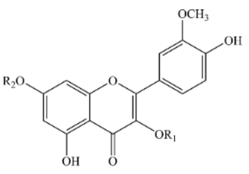

53 R_1=H, R_2=CH_3
54 R_1=β-D-Glc, R_2=α-L-Rha

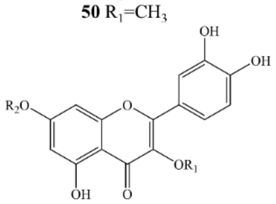

55 R_1=H, R_2=H
56 R_1=α-L-Rha-(1→6)-β-D-Glc, R_2=β-D-Glc
57 R_1=β-D-GlcA, R_2=H
58 R_1=α-L-Rha-(1→6)-β-D-Glc, R_2=H

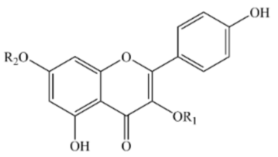

59 R_1=H, R_2=H
60 R_1=β-D-Glc, R_2=α-L-Rha
61 R_1=β-D-Glc-(1→2)-β-D-Glc-(1→2)-β-D-Glc, R_2=H
62 R_1=β-D-GlcA, R_2=H
63 R_1=α-L-Rha, R_2=H
64 R_1=α-L-Rha, R_2=α-L-Rha
65 R_1=β-D-Glc-(1→6)-β-D-Gal, R_2=H

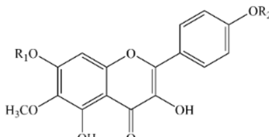

66 R_1=H, R_2=CH_3
67 R_1=CH_3, R_2=H

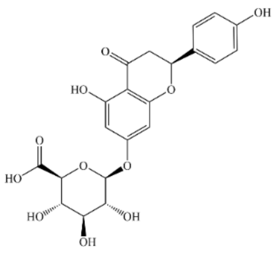

68

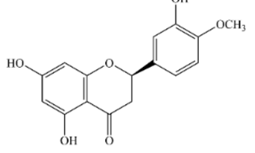

69

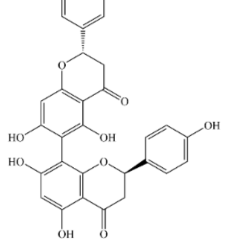

70

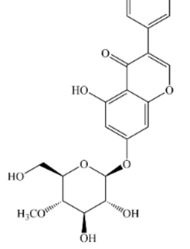

71

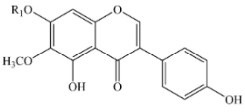

72 R_1= β-D-Glc
73 R_1=H

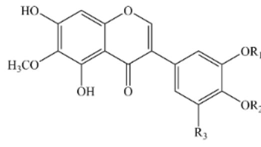

74 R_1=H, R_2=CH_3, R_3=H
75 R_1=CH_3, R_2=H, R_3=H
76 R_1=CH_3, R_2=CH_3, R_3=OH

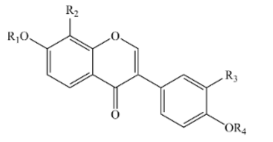

77 R_1=β-D-Glc, R_2=H, R_3=H, R_4=H
78 R_1=H, R_2=H, R_3=H, R_4=CH_3
79 R_1=H, R_2=β-D-Glc, R_3=OH, R_4=H

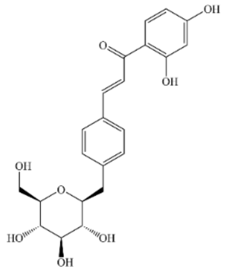

80

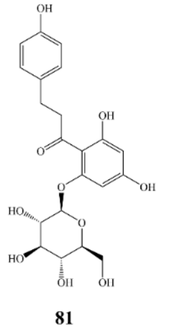

81

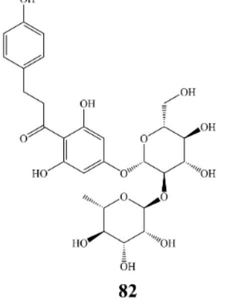

82

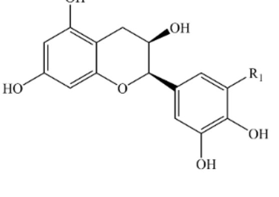

83 R_1=H
84 R_1=OH

Supplemental Figure S2. Structures of other flavonoid subclasses isolated from *O. corniculata*, including flavonols (49–67), dihydroflavones (68–70), isoflavones (71–79), chalcone (80), dihydrochalcones (81–82), and flavanols (83–84).

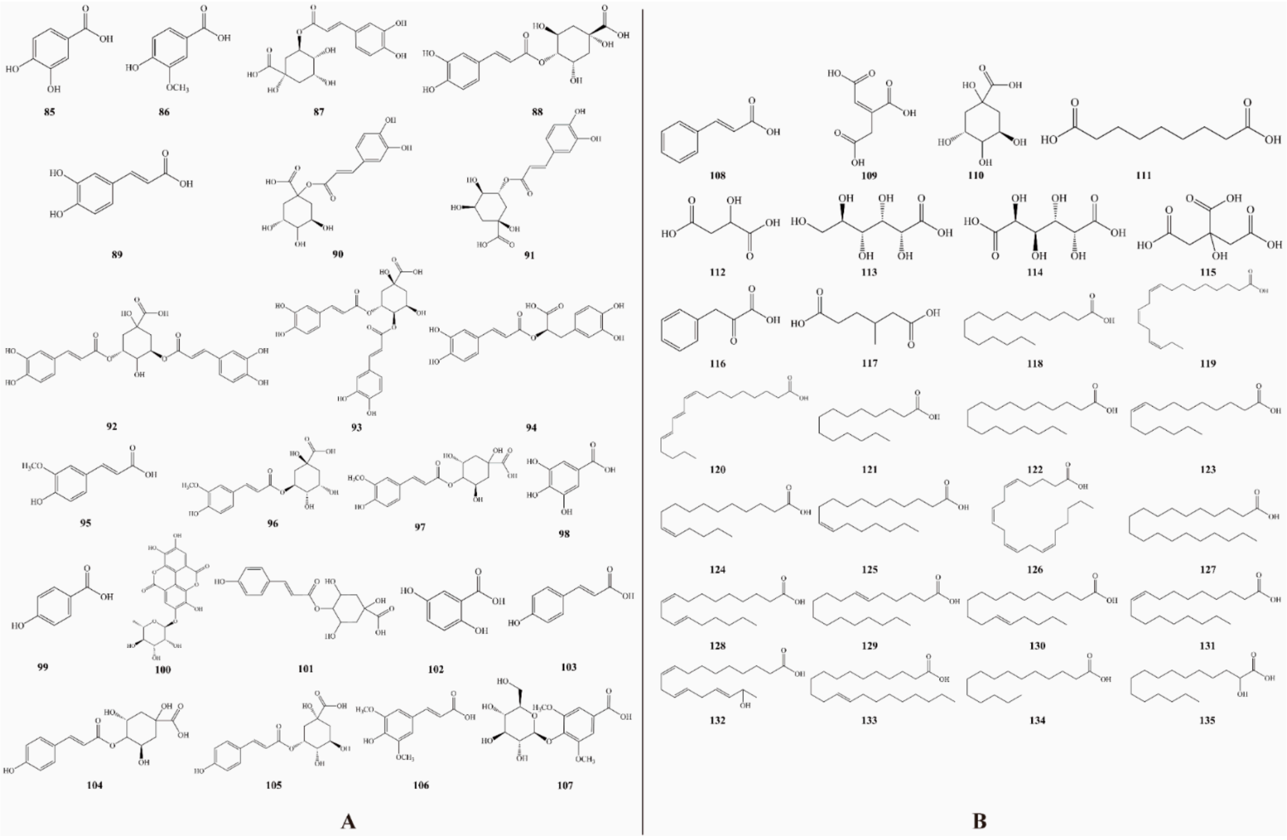

Supplemental Figure S3. Structures of organic acids identified from *O. corniculata*. (A): Phenolic acids (85–107); (B): Other organic acids (108–135).

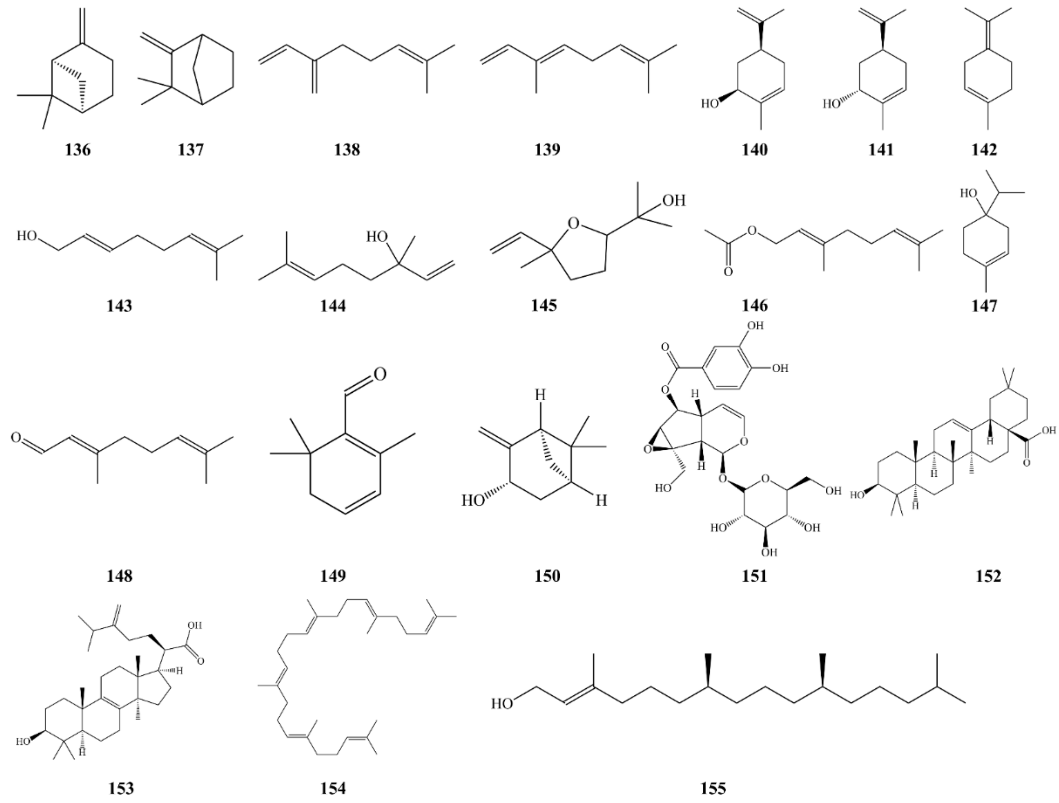

Supplemental Figure S4. Structures of terpenoids (136–155) identified from *O. corniculata*.

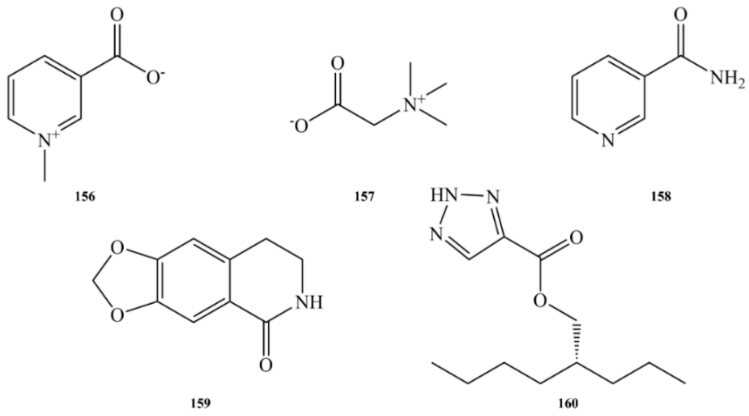

Supplemental Figure S5. Structures of alkaloids identified from *O. corniculata*.

Reference

[1] Bao Z, Weimi K, Jia L I U, Li J, Yongjun L I, Xue M A. Chemical Constituents Analysis of *Oxalis Corniculata* L. Based on UPLC-Q-Exactive-Plus-Orbitrap-MS[J]. *Chinese Journal of Modern Applied Pharmacy*, 2025,42(4):611-622.

[2] Feng Q, Yang W, Peng Z, Wang G. Utilizing bio-affinity ultrafiltration combined with UHPLC Q-Exactive Plus Orbitrap HRMS to detect potential α-glucosidase inhibitors in *Oxalis corniculate* L.[J]. *International Journal of Biological Macromolecules*, 2023,252:126490.

[3] Ibrahim M, Hussain I, Imran M, Hussain N, Hussain A, Mahboob T. Corniculatin A, a new flavonoidal glucoside from *Oxalis corniculata*[J]. *Revista Brasileira de Farmacognosia*, 2013,23(4):630-634.

[4] Abu-Elfotuh K, Hamdan A M E, Mohamed S A, Bakr R O, Ahmed A H, Atwa A M, Hamdan A M, Alanzai A G, Alnahhas R K, Gowifel A M H, Salem M A. The potential anti-Alzheimer's activity of *Oxalis corniculata* Linn. Methanolic extract in experimental rats: Role of APOE4/LRP1, TLR4/NF-κβ/NLRP3, Wnt 3/β-catenin/GSK-3β, autophagy and apoptotic cues[J]. *Journal of Ethnopharmacology*, 2024,324:117731.

[5] Zhang B, Peng X, He Y. Chemical constituents from *Oxalis corniculata*[J]. *J Chin Med Mat*, 2018,41:1883-1886.

[6] Prasad Pandey B, Prakash Pradhan S, Adhikari K. LC-ESI-QTOF-MS for the profiling of the metabolites and in vitro enzymes inhibition activity of Bryophyllum pinnatum and *Oxalis corniculata* collected from Ramechhap District of Nepal[J]. *Chemistry & biodiversity*, 2020,17(6):e2000155.

[7] Kiran K S, Kameshwar V H, Mudnakudu Nagaraju K K, Nagalambika P, Varadaraju K R, Karthik N A, Dugganaboyana G K, Nanjunda Swamy S, Krishna K L, Kumar J R. Diosmin: A Daboia russelii venom PLA_2s inhibitor-purified, and characterized from *Oxalis corniculata* L medicinal plant[J]. *Journal of Ethnopharmacology*, 2024,318:116977.

[8] Mondal S, Talukdar P, Mondal T K. Study of molecular docking to detect antihypertensive phytochemicals of *Oxalis corniculata* Linn. against angiotensin converting enzyme[J]. *World Scientific News*, 2018,110.

[9] Gudasi S, Gharge S, Koli R, Patil K. Antioxidant properties and cytotoxic effects of *Oxalis corniculata* on human Hepatocarcinoma (Hep-G2) cell line: an in vitro and in silico evaluation[J]. *Future Journal of Pharmaceutical Sciences*, 2023,9(1):25.

[10] Zhang J, Shen W, He H. Exploring the action mechanism of *Oxalis corniculata* L. decoction in treating osteoarthritis utilizing liquid chromatography–mass spectrometry technology combined with network pharmacology[J]. *Medicine*, 2024,103(35):e39515.

[11] Zeb A, Imran M. Carotenoids, pigments, phenolic composition and antioxidant activity of *Oxalis corniculata* leaves[J]. *Food bioscience*, 2019,32:100472.

[12] Yang J, Jiang J, Zong D, Peng Y, Chen J, Zhao J. Chemical Composition from *Oxalis corniculata* L., Content of Total Flavonoids in *Oxalis corniculata* L. in Different Harvest Times

[J]. *Guangzhou Chemical Industry*, 2021,49(22):64-65.

[13] Imran M, Irfan A, Ibrahim M, Assiri M A, Khalid N, Ullah S, Al-Sehemi A G. Carbonic anhydrase and cholinesterase inhibitory activities of isolated flavonoids from *Oxalis corniculata* L. and their first-principles investigations[J]. I*ndustrial Crops and Products*, 2020,148:112285.

[14] Ahamad T, Khan M A, Khan M F, Ahmad R, Rahman M A, Siddiqui S. *Oxalis corniculata*-Derived Bioactive Compounds Target Hormone Receptors in Breast Cancer: HPLC-ESI-MS/MS Analysis, Cytotoxicity, and Computational Studies[J]. *ChemistrySelect*, 2025,10(8):e202404547.

[15] Rehman A, Rehman A, Ahmad I. Antibacterial, antifungal, and insecticidal potentials of *Oxalis corniculata* and its isolated compounds[J]. *International journal of analytical chemistry*, 2015,2015(1):842468.

[16] Jain B. An evidence-based ethnomedicinal study on *Oxalis corniculata*: Review of decade study[J]. *International Journal of Green Pharmacy (IJGP)*, 2023,17(1).

[17] Mukherjee S, Pal S, Chakraborty R*, et al*. Biochemical assessment of extract from *Oxalis corniculata* L.: Its role in food preservation, antimicrobial and antioxidative paradigms using in situ and in vitro models[J]. *Indian Journal of Experimental Biology*, 2018,56:230-243.

[18] Badgujar H F, Bora S, Kumar U. Eco-benevolent synthesis of ZnO nanoflowers using *Oxalis corniculata* leaf extract for potential antimicrobial application in agriculture and cosmeceutical[J]. Biocatalysis and Agricultural Biotechnology, 2021,38:102216.

[19] Bordoloi M, Bordoloi P K, Dutta P P, Singh V, Nath S, Narzary B, Bhuyan P D, Rao P G, Barua I C. Studies on some edible herbs: Antioxidant activity, phenolic content, mineral content and antifungal properties[J]. *Journal of Functional Foods*, 2016,23:220-229.

[20] Duc L V, Thi M N, Le Hong D, Le Huong G. Antioxidant Activity, Inhibition of No Production and Cytotoxicity of Chemical Compounds Isolated from *Oxalis corniculata* L.[J]. *Pharmaceutical Chemistry Journal*, 2023,57(3):388-394.

[21] Lei Y, Zhang B, Ma X, Zheng L, Gong Z peng, Wang Y lin, Li Y jun. Chemical constituents from *Oxalis corniculata* L[J]. *Chinese Pharmaceutical Journal*, 2021,56(17):1378-1383.

[22] Kim J H, Hong M, Han J H, Lee H J, Choi D H, Hoang K, Van Dung L, Kwon T, Ahn Y. Antioxidant and Anti-inflammatory Effects of *Oxalis Corniculata* Hot Water Extract[J]. *Korean Journal of Medicinal Crop Science*, 2022,30(6):419-429.

[23] Badwaik H, Singh M, Thakur D, Giri T, Tripathi D. The botany, chemistry, pharmacological and therapeutic application of *Oxalis corniculata* Linn-a review[J]. *International Journal of Phytomedicine*, 2011,3(1):1.

[24] Zhang S, Dong J, Cheng H. Essential oil composition of the leaves of *Oxalis corniculata* from China[J]. *Chemistry of Natural Compounds*, 2018,54:380-381.

[25] Karimzadeh K, Bakhshi N, Ramzanpoor M. Biogenic silver nanoparticles using *Oxalis corniculata* characterization and their clinical implications[J]. *Journal of Drug Delivery Science and Technology*, 2019,54:101263.

[26] Durgawale P P, Hendre A S, Phatak R S. GC/MS characterization, antioxidant and free radical scavenging capacities of methanolic extract of *Oxalis corniculata* LINN: an ayurvedic herb[J]. *Rasayan J Chem*, 2015,8(3):271-278.

[27] Loi V D, Nga D T Q, Huong D T M, Huy N Q. Compounds Isolated from the Ethyl Acetate Fraction of the Aerial Parts of *Oxalis corniculata* L.[J]. *VNU Journal of Science: Medical and Pharmaceutical Sciences*, 2018,34(1).

[28] eng Q, Yang W, Ma X, Peng Z, Wang G. Investigation on the anti-α-glucosidase mechanism of aspergillus triazolate A from *Oxalis corniculata* L.[J]. *International Journal of Biological Macromolecules*, 2024,279:135457.

[29] Zhang B, Jiang L, Ma X, Wang A M, Liu T, Zhou M, Liao S G, Wang Y L, Huang Y, Li Y J. A New arylnaphthalide Lignan from *Oxalis corniculata*[J]. *Natural Product Communications*, 2019,14(9):1934578X19875885.

[30] Dighe S B, Kuchekar B S, Wankhede S B. Analgesic and anti-inflammatory activity of β-sitosterol isolated from leaves of *Oxalis corniculata*[J]. *Int J Pharmacol Res*, 2016,6(03):109-113.

[31] Absar K M B, Rifat H B S M, Das S, Eisha J A, Das T R, Dash P R. Phytochemical and Pharmacological Properties of *Oxalis corniculata*: A Review[J]. *Tropical Journal of Phytochemistry and Pharmaceutical Sciences*, 2024,3(7):364-374.
